# (−)‐Epicatechin Rescues Memory Deficits by Activation of Autophagy in a Mouse Model of Tauopathies

**DOI:** 10.1002/mco2.70144

**Published:** 2025-03-24

**Authors:** Yanqing Wu, Ting Li, Xingjun Jiang, Jianmin Ling, Zaihua Zhao, Jiahui Zhu, Chongyang Chen, Qian Liu, Xifei Yang, Xuefeng Shen, Rong Ma, Gang Li, Gongping Liu

**Affiliations:** ^1^ Department of Neurology Union Hospital Tongji Medical College Huazhong University of Science and Technology Wuhan China; ^2^ Health Management Center Renmin Hospital of Wuhan University Wuhan China; ^3^ Department of Pathophysiology School of Basic Medicine Key Laboratory of Ministry of Education of China and Hubei Province for Neurological Disorders Tongji Medical College Huazhong University of Science and Technology Wuhan China; ^4^ Department of Pathology Renmin Hospital of Wuhan University Wuhan China; ^5^ Department of Neurology The First Affiliated Hospital of Zhengzhou University Zhengzhou China; ^6^ Department of Emergency Medicine Tongji Hospital Tongji Medical College Huazhong University of Science and Technology Wuhan China; ^7^ Department of Critical Care Medicine Tongji Hospital Tongji Medical College Huazhong University of Science and Technology Wuhan China; ^8^ Department of Occupational and Environmental Health and the Ministry of Education Key Lab of Hazard Assessment and Control in Special Operational Environment School of Public Health Air Force Medical University Xi'an China; ^9^ Key Laboratory of Nuclear Medicine Ministry of Health Jiangsu Key Laboratory of Molecular Nuclear Medicine Jiangsu Institute of Nuclear Medicine Wuxi China; ^10^ Key Laboratory of Modern Toxicology of Shenzhen Shenzhen Center for Disease Control and Prevention Shenzhen China; ^11^ Department of Pharmacology School of Basic Medicine Tongji Medical College Huazhong University of Science and Technology Wuhan China; ^12^ Co‐Innovation Center of Neuroregeneration Nantong University Nantong China

**Keywords:** autophagy, (−)‐Epicatechin, FTDP, memory deficits, tauopathies

## Abstract

In tauopathies, defects in autophagy‐lysosomal protein degradation are thought to contribute to the abnormal accumulation of aggregated tau. Recent studies have shown that (−)‐Epicatechin (Epi), a dietary flavonoid belonging to the flavan‐3‐ol subgroup, improves blood flow, modulates metabolic profiles, and prevents oxidative damage. However, less research has explored the effects of Epi on tauopathies. Here, we found that Epi rescued cognitive deficits in P301S tau transgenic mice, a model exhibiting characteristics of tauopathies like frontotemporal dementia and Alzheimer's disease, and attenuated tau pathology through autophagy activation. Proteomic and biochemical analyses revealed that P301S mice exhibit deficits in autophagosome formation via modulating mTOR, consequently inhibiting autophagy. Epi inhibited the mTOR signaling pathway to promote autophagosome formation, which is essential for the clearance of tau aggregation. By using chloroquine (CQ) to inhibit autophagy in vivo, we further confirmed that Epi induced tau degradation via the autophagy pathway. Lastly, Epi administration was also found to improve cognition by reversing spine decrease and neuron loss, as well as attenuating neuroinflammation. Our findings suggest that Epi promoted tau clearance by activating autophagy, indicating its potential as a promising therapeutic candidate for tauopathies.

## Graphical abstract

Accumulated tau induces autophagy deficits by activating mTOR. However, (−)‐Epicatechin enhanced autophagosome formation, leading to the degradation of aggregated tau proteins via suppressing mTOR activity. This activation of autophagy and subsequent reduction in tau pathology contribute to the observed improvements in synaptic density, reversed neuron loss and attenuated neuroinflammation, and enhanced cognitive function in P301S mice. The graphical abstract image was created using Adobe Photoshop CS3.

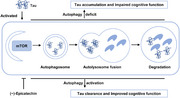

## 1 Introduction

Tauopathies, characterized by the presence of abnormally phosphorylated tau aggregates, are implicated in more than 20 types of age‐related neurodegenerative diseases, including frontotemporal dementia (FTDP‐17), Alzheimer's disease (AD), progressive supranuclear palsy (PSP), and so on [1]. It is estimated more than 42 million people over the age of 60 worldwide have dementia, with the majority likely suffering from tauopathies [2]. Patients affected by tauopathies exhibit progressive cognitive impairments and have a markedly reduced life span as a consequence of various severe psychomotor disorders [3].

Intraneuronal tau accumulations form paired helical filaments (PHFs) and neurofibrillary tangles (NFTs), causing microtubule defects and leading to synapse damage, neuron loss, and microglial activation. Braak staging describes NFT progression in AD [4], with tau aggregation levels correlating with neurodegeneration and memory loss [5]. Transgenic mice rTg4510, carrying the P301L mutation linked to frontotemporal dementia and Parkinsonism linked to chromosome 17 (FTDP‐17), develop NFTs and behavioral issues in an age‐ and gene‐dose‐dependent fashion [6]. Similarly, htau mice overexpressing human tau show brain pathology and cognitive decline [7]. We have reported that tau disrupts mitochondrial dynamics [7], and calcium signaling [7], and inhibits autophagosome‐lysosome fusion by affecting IST1‐regulated ESCRT‐III complex formation [7].

The exact cause of tau aggregation is unknown but may involve protein homeostasis imbalances [8]. Autophagy, crucial for clearing abnormal proteins and damaged organelles in cells, is important for brain health [9]. Its dysfunction is linked to tauopathy progression and is common in AD models [9]. In late‐onset sporadic AD, several AD risk genes, including PICALM, GRN, and BIN1, have been implicated in the dysregulation of autophagy [10]. In neurons of AD brains, the blocked autophagy processes are evidenced by massive accumulation of autophagic vacuoles including autophagosomes and autolysosomes within the dystrophic neuritis [11]. Intracellular tau aggregates can be reduced through the activation of autophagy, using agents such as rapamycin [12], trehalose [13], or methylene blue [14]. Thus, this strong link between autophagy dysfunction and intracellular tau aggregation suggests that restoring or activating autophagy could be a viable therapeutic strategy for tauopathies.

P301S transgenic mice, which carry the P301S mutation in the human tau gene, are widely used as an animal model for FTDP‐17 tauopathies. The mice exhibit ultrastructural and biochemical similarities to human tauopathies in the brain [15], which have been used to investigate novel therapeutic strategies for tau‐related disorders [16]. (−)‐Epicatechin (Epi) is a natural compound of polyphenolic flavonoids, which exists as a monomer in cocoa beans at relatively high concentrations and is known for its neuroprotective, anti‐oxidant, and anti‐apoptotic properties [17]. Epi is capable of crossing the blood‐brain barrier and acting directly on neurons and other brain cells, without significant side effects in humans or mice [18]. Given that Epi‐containing foods have been shown to improve cognitive functions in both human and animal models [19], here, using P301S mice, we investigated the effects of Epi on tau pathology. Proteomic and biochemical analyses revealed that Epi treatment inactivated mTOR signaling, thereby promoting tau degradation. Epi also ameliorated synaptic density decrease, neuron loss, and neuroinflammation. Collectively, these effects contributed to the improvement of cognitive function in P301S mice. Our findings suggest that Epi holds promise as a potential therapeutic candidate for the treatment of tauopathies.

## 2 Results

### 2.1 Epi Treatment Attenuated Cognitive Impairments of P301S Mice

As displayed signs of age‐related spatial memory deficits and tau pathology during 6–9 months old, P301S mice were treated with either Epi (50 mg/kg) or vehicle for 2 months by oral gavage, starting at 7 months of age. Learning and memory ability were evaluated using the novel object recognition (NOR), the Morris water maze (MWM), and the fear condition (FC) test.

**FIGURE 1:**
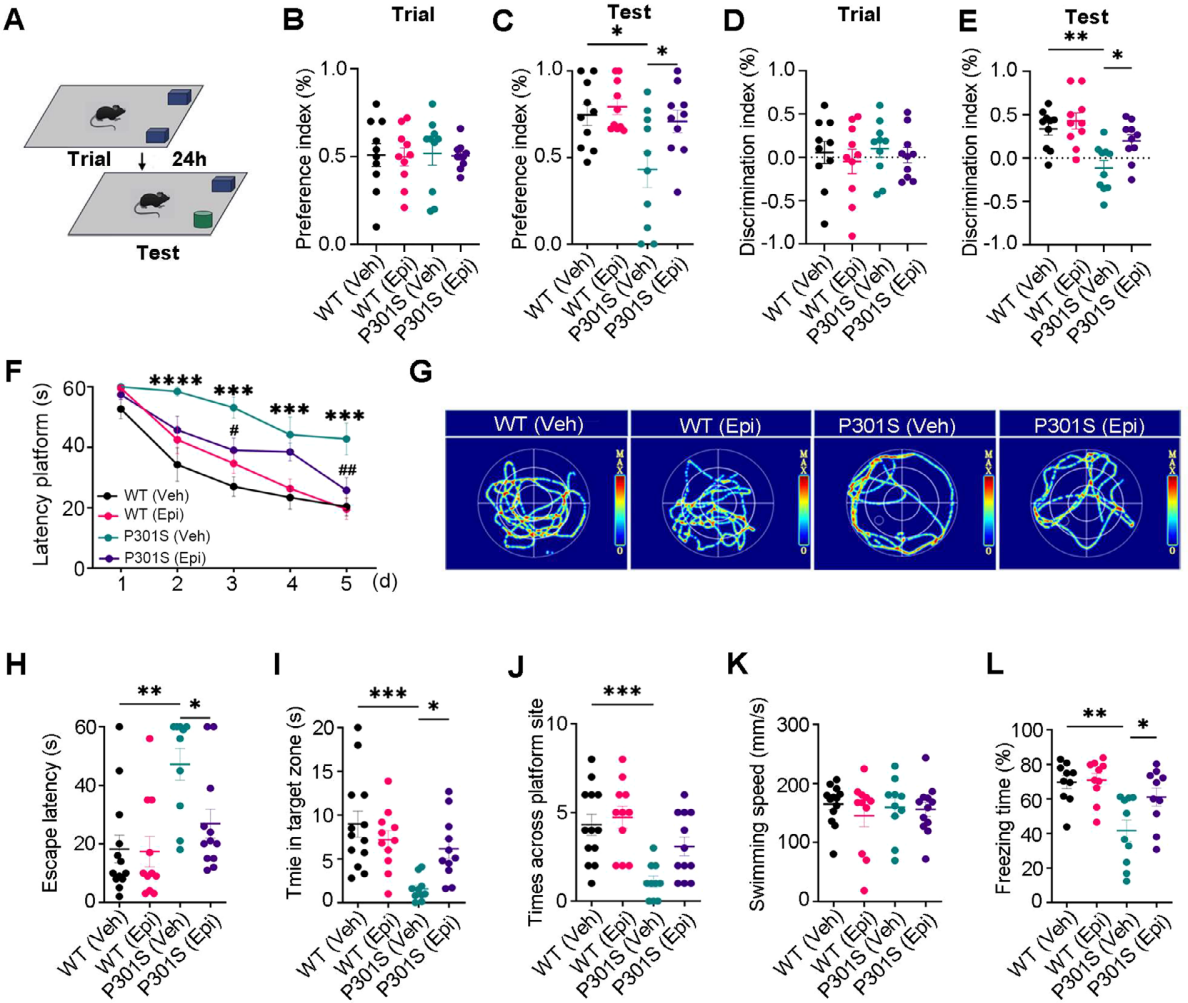
Epi attenuated cognitive impairments in P301S mice. Seven‐month‐old P301S mice and their wild type littermates (WT) were treated with Epi for 2 months, after which their cognitive abilities were assessed. (A) Pattern diagram for new object recognition (NOR). (B–E) Preference or discrimination index during training (B, D) and test (C, E) in NOR (*n* = 10 mice/group). (F) Latency to platform during a 5‐day training period of Morris water maze (MWM), *n* = 10–13 mice/group, *, *p <* 0.05, ***, *p < *0.001, ****, *p < *0.0001 versus WT (Veh); *^#^*, *p <* 0.05, *^##^*, *p <* 0.01 versus P301S (Veh). (G–K) The cognitive ability was detected by MWM. The representative swimming trace of the mice during the test phase of MWM (G), Escape latency (H), time in the target zone (I), and times across the platform (J) of mice. There was no significant difference in swimming speed among four groups (K). (*n* = 10–13 mice/group). (L) The freezing times were detected by fear condition test. (*n* = 10 mice/group). All data are shown as mean ± SEM. Two‐way repeated measures ANOVA test followed by Tukey's post hoc test for F, and One‐way ANOVA test followed by Tukey's post hoc test for others. *, *p <* 0.05, **, p < 0.01 ***, *p < *0.001.

Although transgenic mice demonstrated similar learning performance (preference index and discrimination index) to WT mice on the training day (Figure 1A,B,D), P301S mice spent less time with the novel object, as reflected by the decreased preference and discrimination index compared to WT controls. However, Epi treatment attenuated these deficits in P301S mice (Figure 1C,E). Using the MWM test, impaired learning abilities of P301S mice were evident by the increased escape latency in 2nd–5th days compared to those of the WT controls, however, Epi treatment attenuated the learning deficits of P301S mice at 3rd and 5th days (Figure 1F). Epi administration also ameliorated the memory impairments in P301S mice, as demonstrated by the reduced time (escape latency) to reach the location where the platform was placed previously, and increased time stayed in the target zone than the vehicle (Veh)‐ treated P301S mice, while there was no significant difference in the times across the target zone (Figure 1G–J). No significant difference in swimming speed was seen among the four groups (Figure 1K), which excluded motor deficits. By contextual fear conditioning test, we also observed that Epi treatment rescued memory defects as shown by an increased freezing time during the memory test (Figure 1L), with no differences observed on the training day (Figure S1). These data suggested that Epi treatment ameliorated the cognitive deficits of P301S mice.

### 2.2 Epi‐Attenuated Synaptic Density Decrease, Neuron Loss, and Neuroinflammation in P301S Mice

**FIGURE 2:**
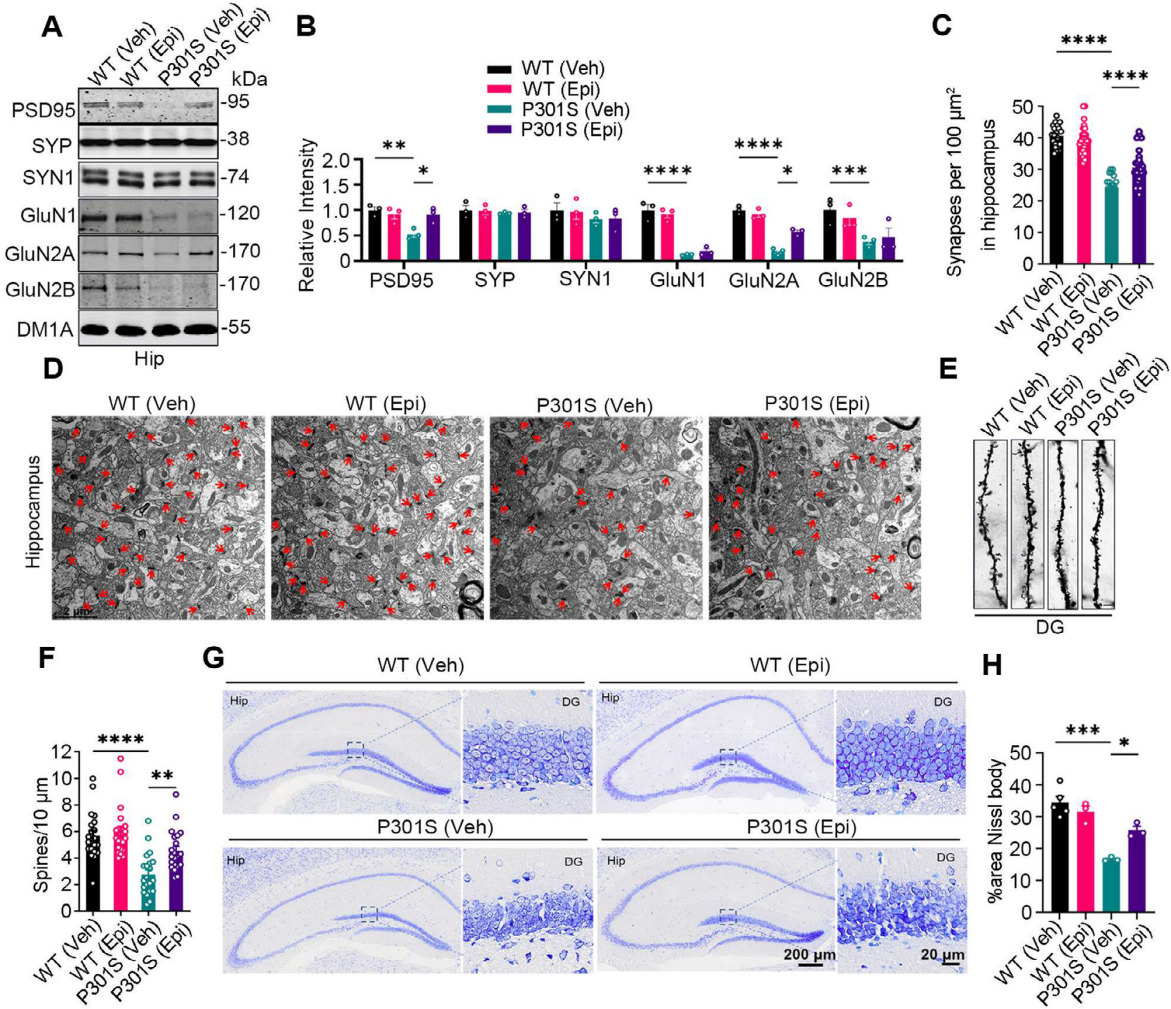
Epi rescued synaptic disorders and neuronal loss in the hippocampus of P301S mice. (A, B) Partial synaptic‐related proteins post synaptic density protein 95 (PSD95), Synaptophysin (SYP), Synapsin‐1 (SYN1), NMDAR1 (GluN1), NMDAR‐2A (GluN2A) and NMDAR‐2B (GluN2B) levels were analyzed by western blotting (*n* = 3 mice for each group). (C, D) The synaptic density in the hippocampus (Hip) of mice were detected by electron microscopy and quantitative analysis. (*n* > 25 from 3 mice for each group). (E, F) Dendritic spine density was detected by Golgi staining and quantitative analysis. Scale Bar, 5µm. (*n* = 21 neurons from 3 mice for each group) (G, H) Representative Nissl staining images in the hippocampus (G) and quantitative analysis (H). (Hip: hippocampus; DG: dentate gyrus. *n* = 3–5 for each group). All data are shown as mean ± SEM. One‐way ANOVA test followed by Tukey's post hoc test, *, *p* < 0.05, **, *p* < 0.01, ***, *p* < 0.001, ****, *p* < 0.0001. WT (Veh): wild type mice treated with vehicle; WT (Epi): wild type mice treated with (−)‐Epicatechin; P301S (Veh): P301S mice treated with vehicle; P301S (Epi): P301S mice treated with (−)‐Epicatechin.

Tau‐mediated synaptic defects, neuron loss, and neuroinflammation are key hallmarks of tauopathies [20]. To determine whether Epi rescues these phenotypes in P301S mice, we assessed the levels of partial pre‐synaptic and post‐synaptic proteins. Epi treatment attenuated the decreases of post‐synaptic proteins, post‐synaptic density protein PSD‐ 95, and NMDA receptor 2A (GluN2A), in the hippocampus of P301S mice, while it had no effects on the post‐synaptic proteins GluN1 and GluN2B, which were reduced in the hippocampus of P301S mice compared to WT mice (Figure 2A,B). No significant differences were observed in the levels of the pre‐synaptic proteins synaptophysin (SYP) and synapsin1 (SYN1) between groups. Electron microscopy revealed a significant loss of synapses in the hippocampus of P301S mice compared to WT controls, which was ameliorated by Epi administration (Figure 2C,D). Golgi staining further demonstrated that Epi rescued the loss of dendritic spine density in the hippocampus of P301S mice compared to P301S controls (Figure 2E,F). In the DG area, the number of neurons was significantly reduced in P301S mice compared to WT controls; however, Epi treatment increased neuronal count in the hippocampus of P301S mice (Figure 2G,H). Together, these findings demonstrated that Epi reversed synapse defects and neuron loss in P301S mice.

Immunohistochemistry revealed a significant increase in IBA1‐ and GFAP‐positive staining in the hippocampus of P301S mice compared to WT controls, which was attenuated by Epi treatment (Figure S2A,B). No substantial changes in IBA1 or GFAP‐positive staining were observed in WT mice treated with vehicle or Epi. We performed qRT‐PCR to examine inflammatory factors. Interleukins (IL‐1α, ‐1β, ‐2, ‐6) and TNF‐α were significantly elevated in the hippocampus or cortex of P301S mice compared to WT controls. Epi treatment reduced the levels of these inflammatory factors in the hippocampus, as well as IL‐1β, ‐2, and ‐6 in the cortex of P301S mice (Figure S2C,D). These findings suggested that Epi treatment attenuated neuroinflammation in the brains of P301S mice.

### 2.3 Epi‐Promoted Tau Protein Levels Degradation In Vivo

**FIGURE 3:**
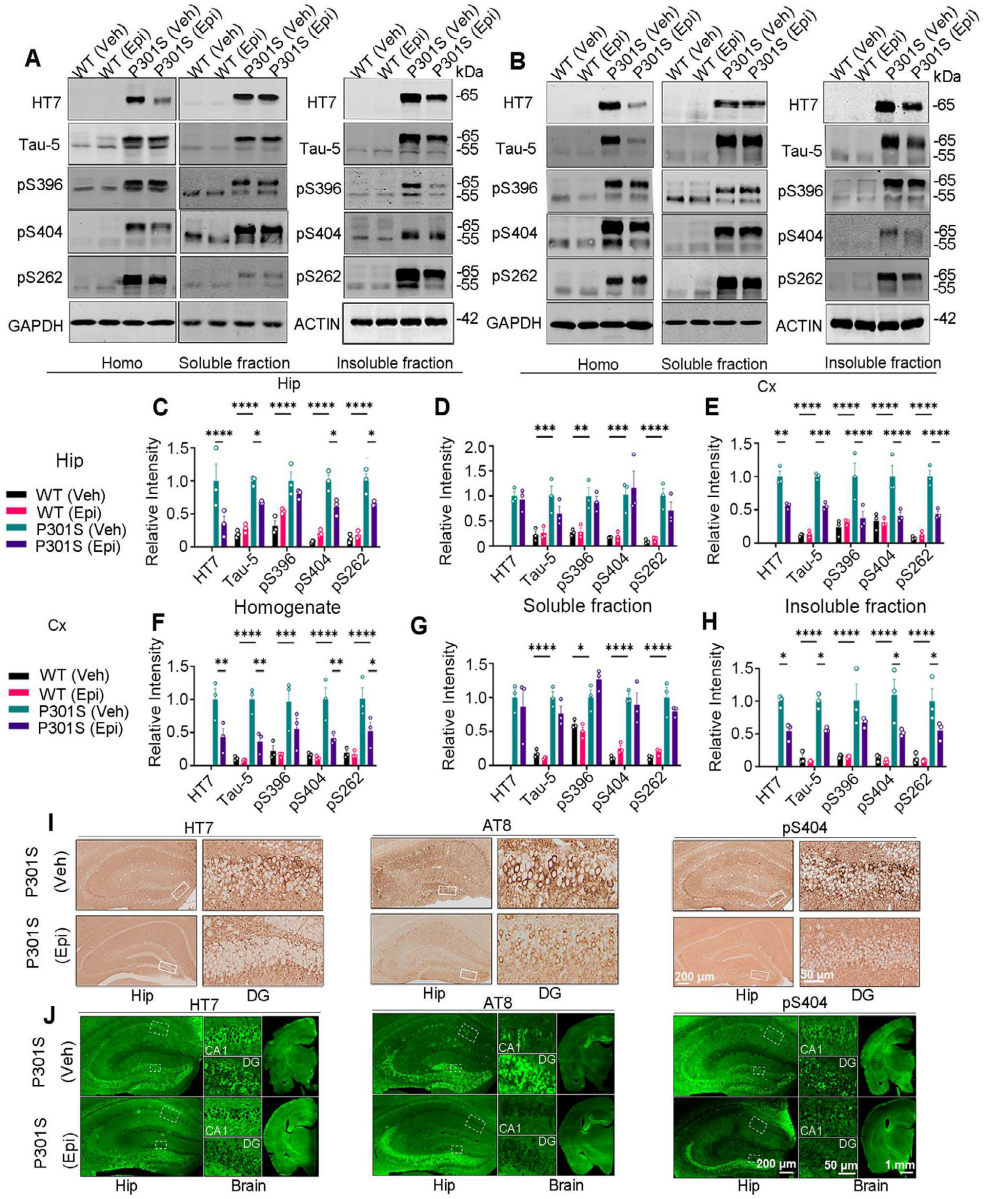
Epi treatment reduced tau levels in the hippocampus and cortex of P301S mice. Seven‐month‐old P301S mice and the wild type littermates were treated with Epi or vehicle (Veh) for 2 months. Epi treatment reduced total tau (recognized by HT7 or Tau‐5) and phosphorylated tau (pS396, pS404, and pS262) levels in the whole homogenate, or insoluble fraction of the hippocampus (Hip) (A), or cortex (Cx) (B) of P301S mice, which detected by western blotting. Total (recognized by HT7 or Tau‐5) and phosphorylated tau (pS396, pS404 and pS262) level in the whole homogenate (A, C and B, F), soluble (A, D and B, G) or insoluble fraction (A, E and B, H) of the hippocampus (A, C–E), or cortex (B, F–H) were quantitative analysis (*n* = 3 mice for each group). (I, J) Representative immunohistochemical (I) or immunofluorescent images (J) of total tau protein (HT7), phospho‐Tau (AT8, pS404) in the hippocampus of P301S mice (DG: dentate gyrus; Hip: hippocampus). *, *p <* 0.05, **, *p <* 0.01, ***, *p < *0.001, ****, *p < *0.0001. All data are shown as mean ± SEM. One‐way ANOVA test followed by Tukey's post hoc test or Unpaired Student's *t*‐test. Hippocampus (Hip), cortex (Cx).

Next, we assessed tau levels in the homogenate (total), soluble, and insoluble fractions from brain tissue. Western blotting analysis revealed a significant reduction in total tau (recognized by HT7 and Tau‐5 antibodies), and phosphorylated tau (pS404 and pS262) in the whole homogenate of the hippocampus and cortex following Epi treatment, with the exception of pS396, which remained unchanged (Figure 3A,C and B,F). Epi administration significantly reduced total tau, and phosphorylated tau (pS396, pS404, and pS262) in the insoluble fractions of the hippocampus, and total tau, pS404, and pS262 in the insoluble fractions of the cortex (Figure 3A,E and B,H). Epi treatment did not significantly affect the levels of total or phosphorylated tau in the soluble fractions of the hippocampus and cortex (Figure 3A,D and B,G). Immunohistochemical and immunofluorescent staining revealed that Epi treatment significantly reversed the immuno‐positive staining of total tau (HT7) and phosphorylated tau at Ser202, Thr 205 (AT8), and Ser404 (pS404) in the hippocampus of P301S mice, with no significant difference in the pS404 of WT mice with Epi treatment or not (Figure 3I,J and Figure S3A–D). P301S mice treated with Epi also exhibited reduced Thioflavin S‐positive staining in the piriform cortex compared to vehicle‐treated P301S mice (Figure S4). Epi treatment reduced NFTs in the piriform cortex of P301S mice, as evidenced by silver staining (Figure S5). To assess the specificity of Epi's effect on tau protein reduction, we measured the levels of other structural proteins. No alterations in microtubule‐associated protein 2 (MAP2) and TAR DNA binding protein‐43 (TDP43) levels were observed in Epi‐treated P301S mice (Figure S6A,B). We also found that Epi treatment did not significantly alter tau mRNA levels in the cortex and hippocampus of P301S mice (Figure S7A,B). Overall, these findings suggested that Epi treatment significantly reduced accumulated tau levels in vivo.

### 2.4 Epi‐Reduced Tau Protein Levels via Autophagy Pathway In Vitro

**FIGURE 4:**
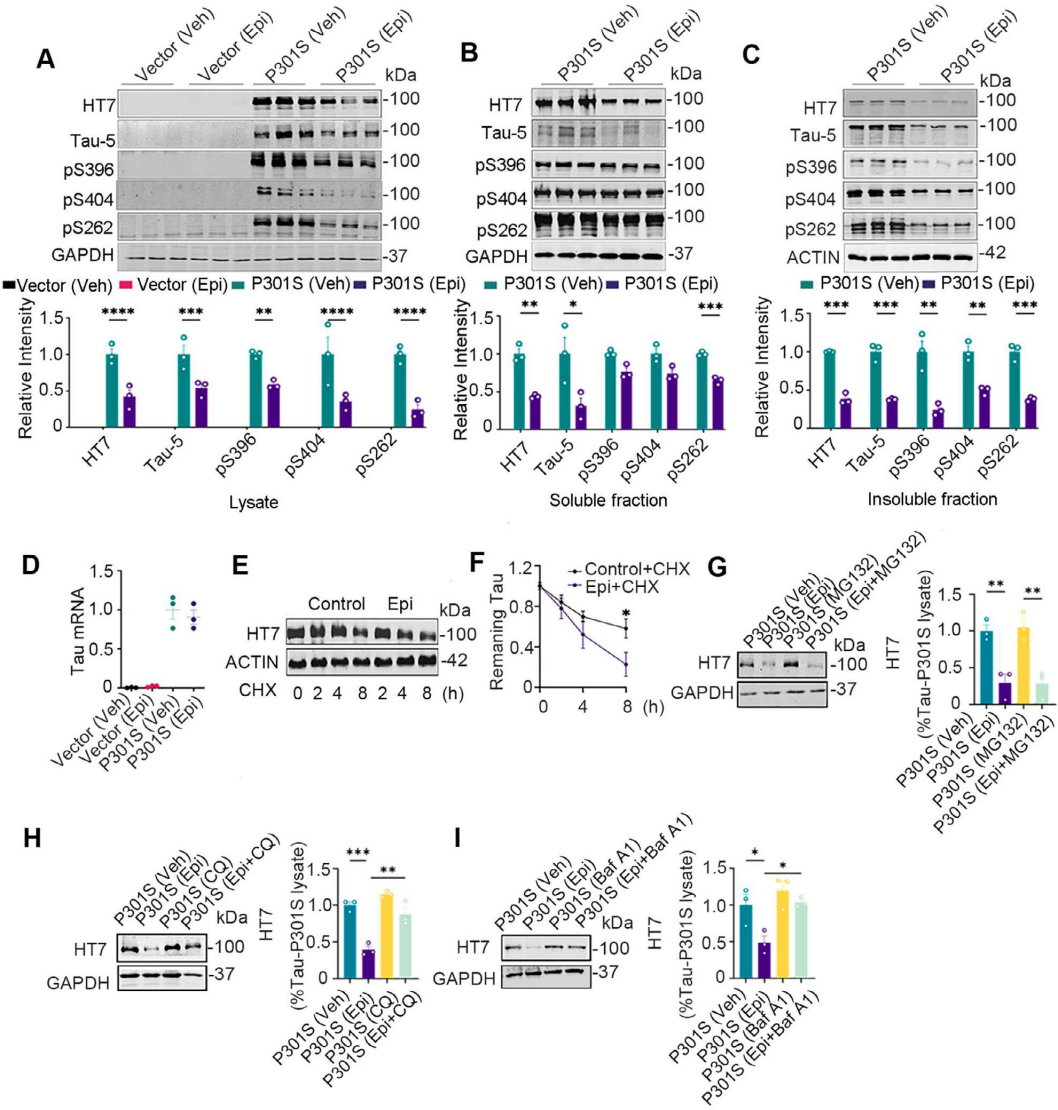
Epi reduced tau level via autophagy in vitro. (A–C) Epi was treated into HEK293/P301S cells for 24 h, total tau (recognized with HT7 and Tau‐5) and phosphorylated tau (pS396, pS404, and pS262) in the whole lysate (A), soluble (B) or insoluble fraction (C) were detected by western blotting. (D) Epi (100 µM) treatment had no effects in the mRNA level of tau in HEK293/P301S cells. (E, F) After HEK293 cells were transfected with pEGFP‐C1‐P301S‐tau plasmid for 24 h, Epi (100 µM) or vehicle (DMSO) treated into the cells with cycloheximide (CHX, 100 µg/mL) for indicated times, and total tau level were detected by western blotting and quantitative analysis (*n* = 4). (G–I) After HEK293 cells were transfected with P301S‐tau plasmid for 24 h, Epi (100 µM) were treated to cells with MG132 (20 µM) for 6 h (G), CQ (40 µM) (H) or Baf A1 (100 µM) for 24 h (I), total tau level was detected by western blotting and quantitative analysis (*n* = 3). All data are shown as mean ± SEM. One‐way ANOVA test followed by Tukey's post hoc test. *, *p* < 0.05, **, *p* < 0.01, ***, *p* < 0.001, ****, *p* < 0.0001.

Building on the previous findings, we further explored the effects of Epi on tau protein in vitro. Wild‐type HEK293 cells transiently transfected with pEGFP‐P301S‐htau plasmid (HEK293/P301S) were used to verify the dosage and administration time of Epi in vitro. When treated with 0, 50, 100, or 200 µM Epi, total tau protein levels decreased in a dose‐dependent manner (Figure S8A,B,C), with an EC50 for Epi approximately 85 µM. with 100 µM Epi for 0, 12, 24, and 48 h, total tau protein levels showed a time‐dependent decline (Figure S8D,E). Therefore, we selected a concentration of 100 µM and a 24‐h treatment period for subsequent in vitro experiments. Next, tau levels were measured after HEK293/P301S cells (HEK293 cells transiently transfected with P301S‐tau) were treated with 100 µM Epi for 24 h. We observed that total tau and phosphorylated tau at Ser396, Ser404, and Ser262 (pS396, pS404, and pS262) decreased in the whole cell lysate and the insoluble fraction, and total tau and pS262 in the soluble fraction decreased (Figure 4A–C). Intracellular tau protein levels are regulated by both protein synthesis and degradation. To investigate the underlying mechanisms of Epi‐induced tau protein reduction, we first measured tau mRNA level using qRT‐PCR and found that Epi treatment did not significantly affect tau mRNA level in vitro (Figure 4D). Next, we assessed tau turnover and observed that Epi treatment enhanced tau turnover in the cycloheximide (CHX) chase experiment (Figure 4E,F). The autophagy inhibitor chloroquine (CQ) and Bafilomycin A1 (Baf A1), but not the proteasome inhibitor MG132 reversed Epi‐induced reduction in tau protein levels (Figure 4G–I), suggesting that Epi promoted tau degradation via the autophagy pathway.

### 2.5 Epi Treatment Activated Autophagy by Increasing Autophagosome Formation In Vitro and In Vivo

**FIGURE 5:**
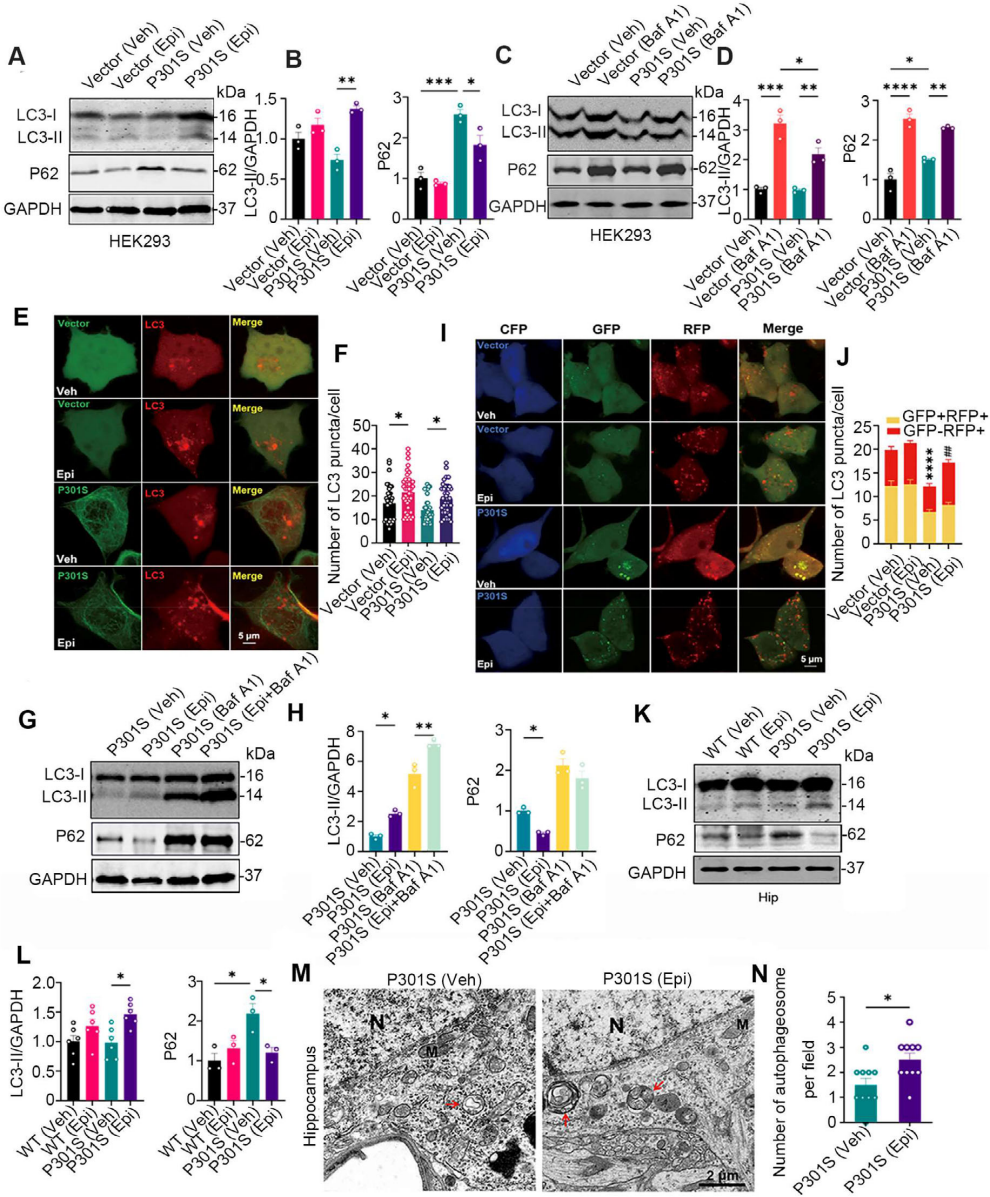
Epi treatment promoted autophagosome formation and restored autophagic flux in P301S tau models. (A, B) After HEK293/Vec or HEK293/P301S cells were treated with Epi for 24 h, the levels of LC3‐II and P62 were detected and quantitative analysis (*n* = 3). (C, D) HEK293/Vec or HEK293/P301S cells were treated with Baf A1 (100 µM) for 24 h, LC3‐II or P62 level was detected by western blotting and quantitative analysis (*n* = 3). (E, F) HEK293 cells co‐transfected with mCherry‐LC3 and EGFP‐C1‐P301S‐tau or the vector (EGFP‐C1) were treated with Epi (100 µM) or vehicle (DMSO) for 24 h, and the mCherry‐LC3 puncta were measured by direct fluorescence imaging (at least 30 cells were analyzed for each group). (G, H) HEK293/P301S cells were treated with Epi (100 µM), Baf A1 (100 µM) or Epi and Baf A1 for 24 h, LC3‐II and P62 levels were detected by western blotting (*n* = 3 for each group). (I, J) The effect of Epi in autophagic flux was evaluated in HEK293 cells transfected with ECFP‐C1‐P301S‐tau or the vector (CFP‐C1) and GFP‐RFP‐LC3 (at least 30 cells were analyzed for each group, ****, *p<*0.0001 vs. Vector (Veh) of GFP^+^RFP^+^ puncta; *^##^*, *p <* 0.01 vs. P301S (Veh) of GFP^−^RFP^+^ puncta). P301S mice and the wild type littermates (WT) at 7 months of age were treated with (−)‐Epicatechin (Epi) or vehicle (Veh) for 2 months. (K, L) LC3‐II or P62 level in the hippocampus (Hip) of WT or P301S mice was detected by western blotting and quantitative analysis (*n* = 3–6 for each group). (M, N) Epi increased autophagosome number in the hippocampal neurons of P301S mice measured by electron microscopy (red arrow, autophagosome; N, nucleus; M, Mitochondria. *n* = 3 mice for each group). Data are expressed as mean ± SEM. *, *p <* 0.05, **, *p <* 0.01, ***, *p < *0.001, ****, *p < *0.0001. One‐way ANOVA test followed by Tukey's post hoc test for the others.

To further confirm that Epi enhances autophagy, HEK293/P301S cells were treated with Epi or Baf A1, followed by the detection of LC3‐II and P62 levels using western blotting. The number of LC3‐positive puncta was analyzed by transfecting cells with the mCherry‐LC3 plasmid and observing through direct fluorescence imaging. Compared to the HEK293/Vec control, the P62 level, but not the LC3‐II level, was elevated in HEK293/P301S (Figure 5A,B). Upon treatment with Baf A1, a specific inhibitor that blocks the fusion of autophagosomes with lysosomes, we observed a marked increase in LC3‐II level and LC3‐positive puncta in HEK293/Vec or HEK293/P301S cells, and the degree of the increase LC3‐II or puncta number in HEK293/Vec was significantly higher than the degree in HEK293/P301S, which suggested that there were obstacles in autophagosome number/formation in HEK293/P301S cells (Figure 5C,D and Figure S9A,B compared vehicle vs. Baf A1). However, in Epi‐treated HEK293/P301S cells, we observed increased LC3‐II levels and LC3‐positive puncta, along with decreased P62 levels (Figure 5A,B,E,F). Combined with Baf A1, Epi further increased LC3‐II level and LC3‐positive puncta in HEK293/P301S (Figure 5G,H and Figure S9C,D), confirming a higher number/formation of autophagosomes induced by Epi. Together, these data demonstrated that P301S‐tau accumulation induced autophagosome formation impairments and Epi treatment increased autophagosome formation.

We further investigated the effects of Epi on autophagic flux by using a double‐tagged LC3 (GFP‐RFP‐LC3) plasmid, which contains an acid‐sensitive GFP and an acid‐insensitive RFP. The transition from autophagosome (neutral pH) to autolysosome (acidic pH) can be visualized by imaging the quench of GFP fluorescence, leaving only red fluorescence. HEK293 cells were transiently co‐expressed with a tandem GFP‐RFP‐LC3 fusion protein and CFP‐C1‐P301S‐tau or CFP‐C1, and the numbers of yellow puncta (GFP^+^RFP^+^, autophagosomes) or red puncta (GFP^−^RFP^+^, mature autolysosomes) were measured using confocal microscopy. Cells treated with Epi exhibited a statistically significant increase in the number of autolysosomes (red dots), indicating a higher rate of autophagic flux (Figure 5I,J).

Furthermore, we found that the P62 level was elevated while the LC3‐II level remained unchanged in the hippocampus of P301S mice compared to WT controls. However, Epi treatment increased the LC3‐II level and decreased the P62 level (Figure 5K,L). Electron microscopy further revealed an increased number of autophagosomes in the hippocampus of P301S mice following Epi treatment (Figure 5M,N). These data together demonstrated that Epi treatment induced autophagosome formation.

### 2.6 mTOR Signaling Pathway Was a Candidate Mechanism for Epi‐Regulated Autophagy

**FIGURE 6:**
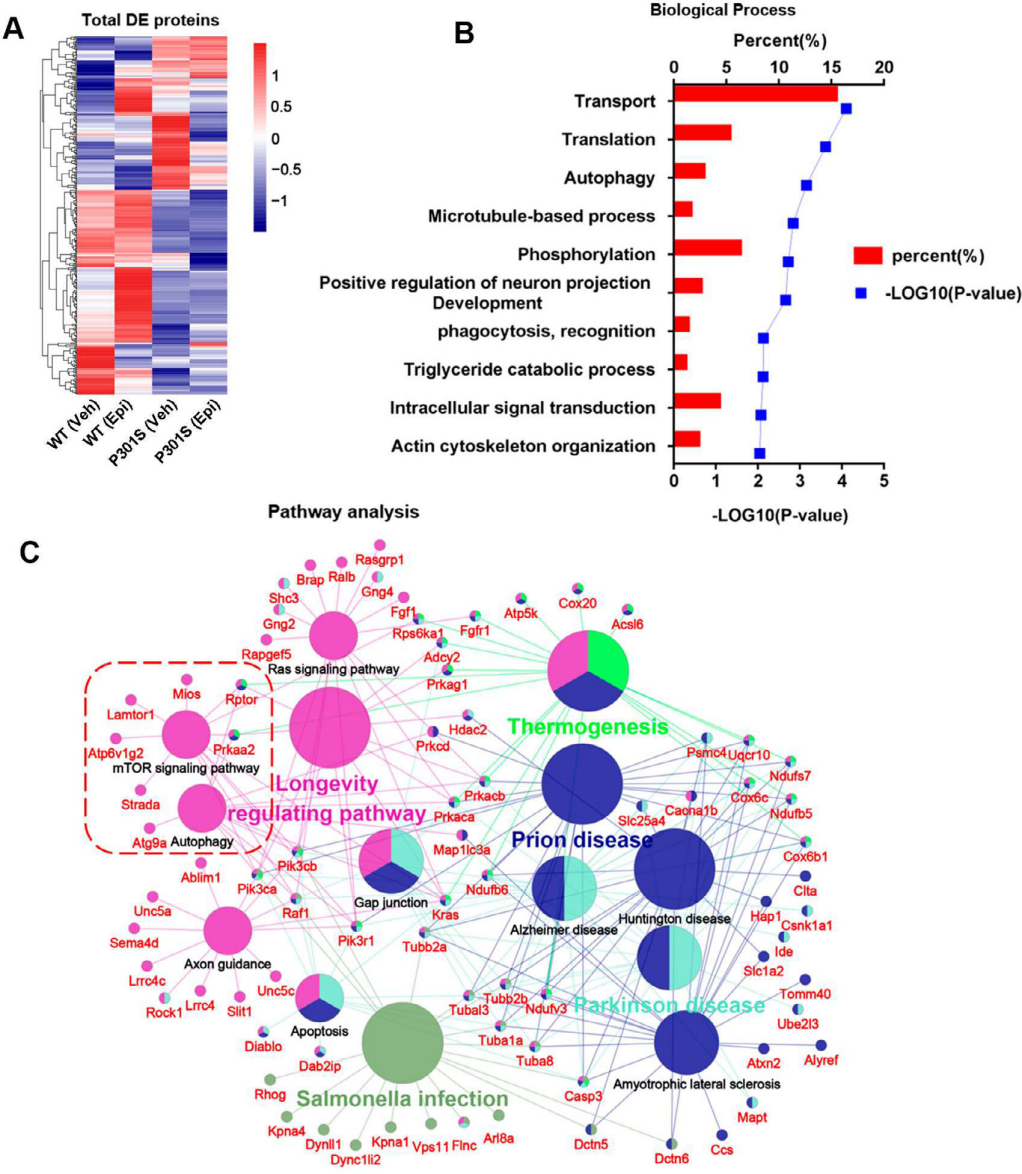
Proteomic analysis of the hippocampus of P301S mice after Epi treatment. (A) Heat map showed the abundance change of total differentially expressed (DE) proteins, which was analyzed in by RStudio. The red indicated higher abundance and blue indicated low abundance. (B) Gene ontology analyzed the total DE proteins to find the critical biological process, which was accomplished in the DAVID (Database for Annotation, Visualization, and Integrated Discovery). The enriched *p* value less than 0.05 as meaningful biological process and the top 10 biological process were list. (C) Pathway analysis of all the DE proteins by using ClueGO plugged into Cytoscape software, each pathway enriched *p* < 0.05 and the minimal gene set was set 7 and the DE proteins were marked red. *n* = 4 mice/group.

We employed an unbiased proteomics approach to identify the potential mechanisms of Epi action. Notably, a total of 404 differentially expressed (DE) proteins were identified in the comparisons between P301S (Veh) versus WT (Veh) and P301S (Epi) versus P301S (Veh). The heatmap indicated that Epi treatment partially reversed the protein expression profile (Figure 6A). Subsequent gene ontology analysis revealed that the biological processes of DE proteins were highly enriched in transport, translation autophagy (*p* value 0.001), etc. (Figure 6B). Pathway analysis further showed that the DE proteins were enriched in pathways related to longevity regulating pathways, salmonella infection, Parkinson's disease, Prion disease, AD, autophagy‐related pathways (mTOR signaling pathway, Autophagy), etc. (Figure 6C). Autophagy activation is well known for attenuating tau pathology and improving cognitive function. Moreover, our experimental results suggested that Epi induced tau degradation via the autophagy pathway. It was assumed that the mTOR signaling pathway was a candidate mechanism for Epi‐regulated autophagy.

### 2.7 mTOR Signal Pathway Was Inactivated by Epi

**FIGURE 7:**
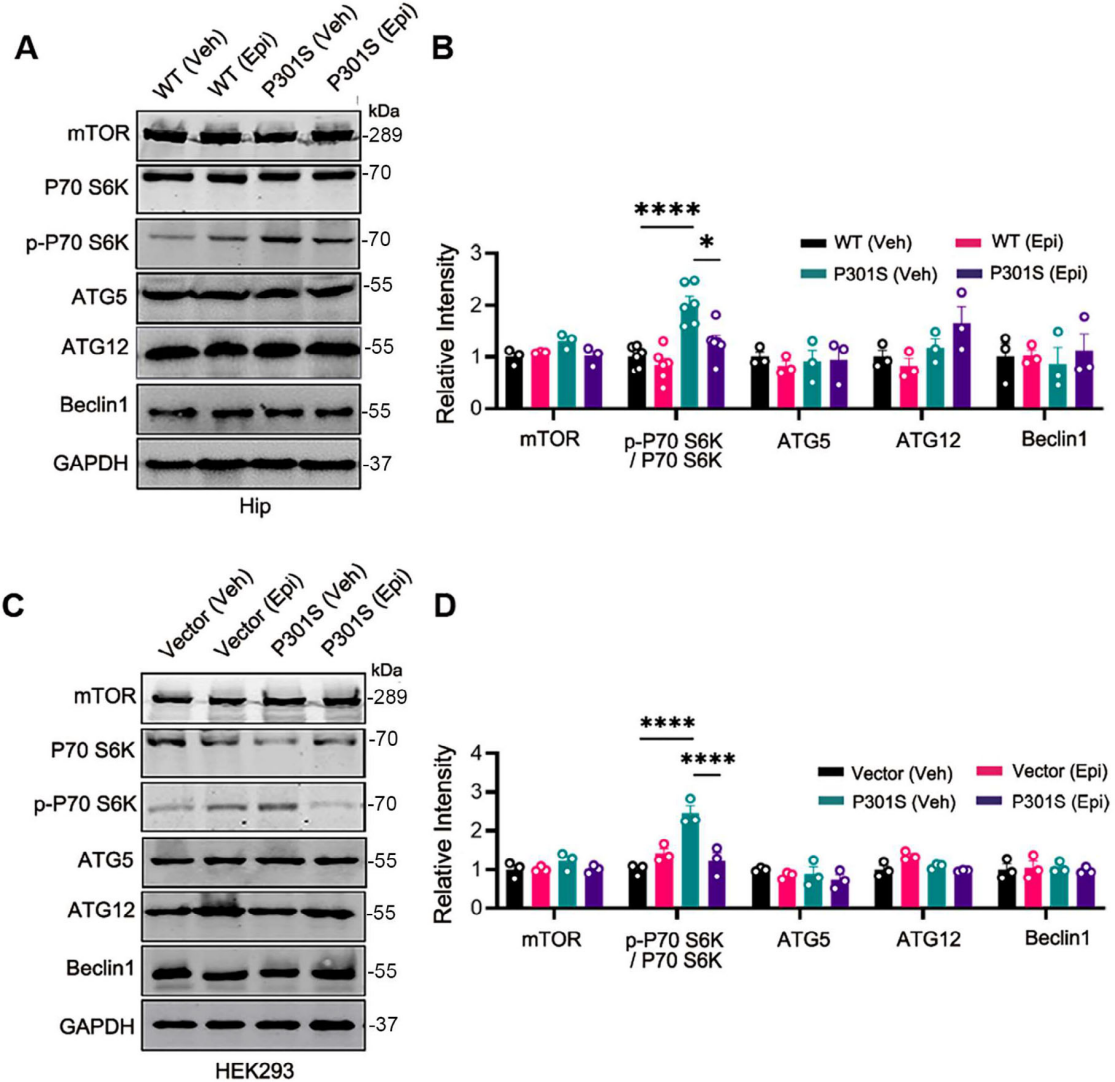
Epi treatment inactivated mTOR. (A, B) WT and P301S mice were treated with Epi for 2 months, hippocampi (Hip) were lysed and phosphorylated P70 S6K (p‐P70 S6K), total P70 S6K, mTOR, and autophagic related proteins were detected by western blotting. (C, D) Western blotting exhibited a decreased level of p‐P70 S6K in HEK293/P301S cells after Epi treatment. All data are shown as mean ± SEM, *n* = 3–6 for each group. One‐way ANOVA test followed by Tukey's post hoc test. *, *p <* 0.05, ***, *p < *0.001, ****, *p < *0.0001.

To validate the proteomics results, we examined the mTOR signaling pathway both in vivo and in vitro using western blotting. Our findings revealed that in comparison to WT control mice, the p‐P70 S6K/ P70 S6K ratio was elevated in the hippocampus of P301S mice. However, treatment with Epi significantly reduced the p‐P70 S6K/ P70 S6K ratio in P301S mice, while the total levels of P70 S6K and mTOR protein remained unchanged (Figure 7A,B). Given that P70 S6K is a downstream target of mTOR and its phosphorylation status reflects mTOR activity [21], these results suggested that Epi inhibited the mTOR signaling pathway, corroborating our proteomic data. We also observed that Epi decreased the level of p‐4EBP1 in P301S mice, which is a phosphorylation target of mTORC1 (Figure S10A,B). Raptor (regulatory‐associated protein of mTOR), a crucial component of mTORC1 that facilitates substrate recruitment and activation of mTORC1 [22], was also found to be reduced in Epi‐treated P301S mice (Figure S10A,B). These findings supported the inhibitory effect of Epi on the mTOR pathway. However, the levels of autophagy‐related protein ATG5, ATG12, and Beclin1, which are involved in autophagosome formation, did not show significant changes in the hippocampus of P301S mice treated with Epi or vehicle (Figure 7A,B). Consistent with the in vivo data, overexpression of P301S‐Tau led to an increase in the p‐P70 S6K/ P70 S6K ratio, whereas treatment with Epi reduced the P301S‐htau‐induced elevation of p‐P70 S6K/ P70 S6K ratio in HEK293/P301S cells. In addition, there were no significant changes observed in other autophagy‐related proteins (Figure 7C,D). Collectively, our findings suggested that Epi modulated the mTOR signaling pathway and promoted autophagy.

### 2.8 Inhibiting Autophagy Reversed the Effects of Epi on Cognition Improvement and Tau Degradation in P301S Mice

**FIGURE 8:**
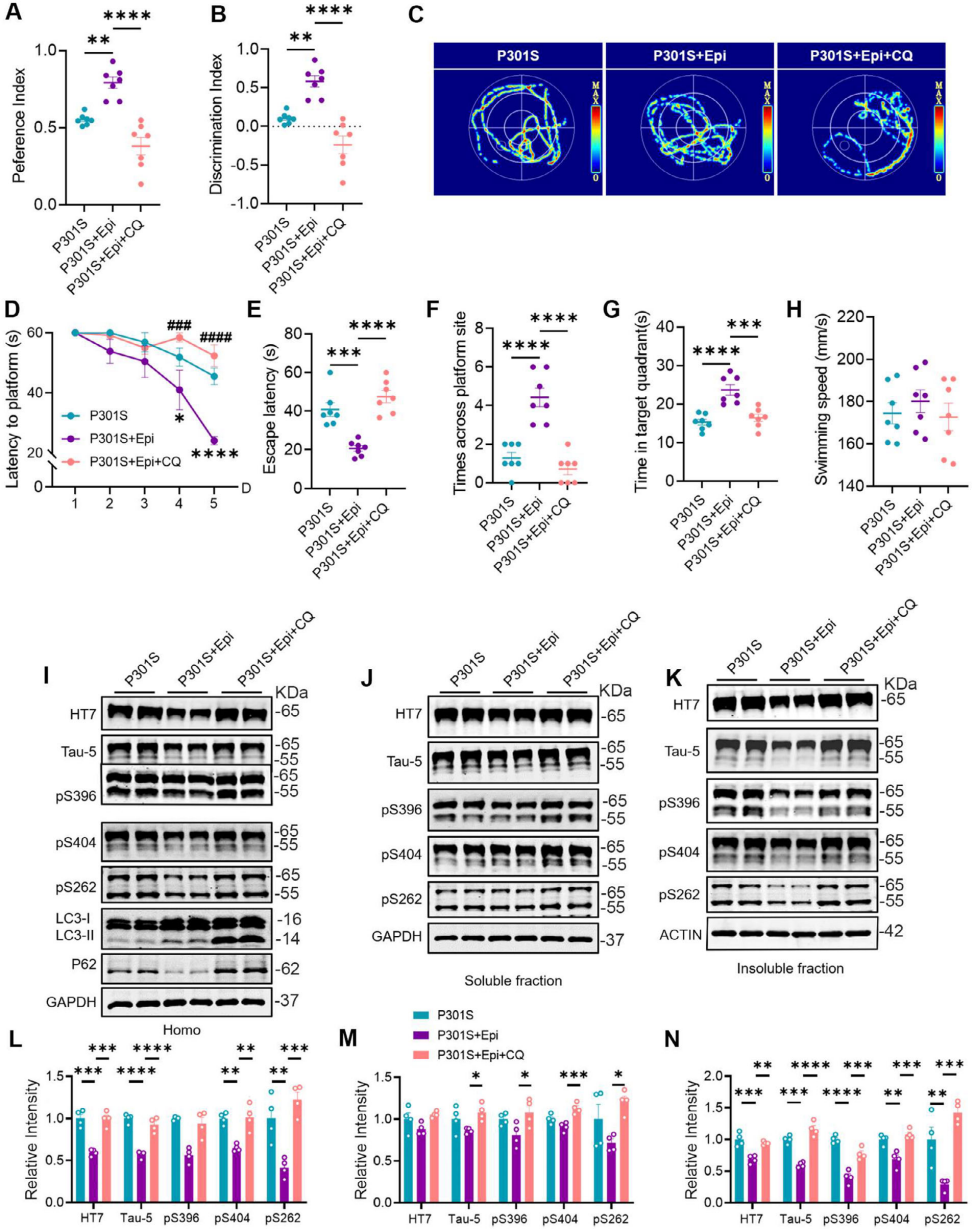
CQ abolished the ameliorated effects of Epi in P301S mice. (A–B) Preference and discrimination index during test in NOR. (C–H) The cognitive ability was detected by Morris water maze (MWM). The representative swimming trace of the mice during the test phase of MWM (C), latency to platform during a 5‐day training period of MWM (D). *, *p* < 0.05, ****, *p* < 0.0001 versus P301S (Veh); ###, *p* < 0.001, ####, *p* < 0.0001 versus P301S + Epi, escape latency (E), time in the target zone (F), and times across the platform (G) of mice. There was no significant difference in swimming speed among four groups (H). *n* = 10–13 mice/group. (I–N), CQ treatment increased total tau (recognized by HT7 or Tau‐5) and phosphorylated tau (pS396, pS404, and pS262) levels in the whole homogenate (I, L), soluble (J, M), and insoluble fraction (K, N) of the hippocampus (Hip) (I–K) of P301S+Epi mice, which detected by western blotting and quantitative analysis. All data are shown as mean ± SEM. Two‐way repeated measures ANOVA test followed by Tukey's post hoc test for F, and One‐way ANOVA test followed by Tukey's post hoc test for others. *, *p *< 0.05, **, *p* < 0.01, ***, *p* < 0.001, ****, *p* < 0.0001.

Furthermore, we conducted behavioral experiments to assess whether the cognitive improvements induced by Epi were also reversed, upon autophagy inhibition. Our results indicated that P301S + Epi mice exhibited a greater amount of time interacting with the novel object, as evidenced by an increased preference and discrimination index when compared to P301S mice. Conversely, CQ treatment resulted in a reduction of these indices in P301S + Epi mice (Figure 8A,B). Utilizing the MWM test, P301S + Epi mice demonstrated enhanced learning abilities, indicated by a decreased escape latency on the 4th and 5th days relative to P301S mice. However, CQ treatment exacerbated the learning deficits of P301S + Epi mice on the 4th and 5th day (Figure 8D). In addition, our findings revealed that CQ administration negated the memory improvements observed in P301S + Epi mice, as evidenced by increased time (escape latency) to reach the site where the platform was placed previously, reduced times across the platform site, and decreased time stayed in the target zone. (Figure 8C,E–G). In addition, there was no significant difference in swimming speed among the four groups (Figure 8H). Subsequently, we assessed tau levels in the homogenate (total), soluble, and insoluble fractions from hippocampal tissue. By western blotting, we observed a significant accumulation of total tau and phosphorylated tau in the hippocampus induced by CQ treatment (Figure 8I–N). These findings suggested that CQ treatment counteracted the beneficial effects on cognition and tau accumulation in P301S mice induced by Epi treatment.

## 3 Discussion

Epi, a polyphenol found in cocoa and tea, increases blood flow, modifies metabolism, and protects against oxidative damage [23]. Moreover, it can cross the blood‐brain barrier, offering neuroprotection [24]. In the present study, we found that Epi administration attenuated tau aggregation in the brains of P301S mice through the activation of autophagy. P301S mice exhibited deficits in the early stages of autophagy due to activated mTOR signaling. In contrast, Epi inhibited the mTOR signaling pathway, promoting autophagosome formation. Furthermore, by blocking the fusion of autophagosomes and lysosomes with CQ, we confirmed that Epi‐induced tau degradation via the autophagy pathway.

Autophagy has been recognized as an important regulator in the degradation of aggregated tau, and elevated autophagic activity is beneficial for tau degradation and tauopathy amelioration [11]. Some studies have found that intake of Epi may provide protection against neurodegenerative diseases [25, 26]. However, the underlying mechanisms remain unknown. Through proteomics, we identified a total of 404 DE proteins in a compared group of P301S (Veh) versus WT (Veh) and a compared group of P301S (Epi) versus P301S (Veh). The enriched pathways among the DE proteins in the P301S versus WT comparison included the mTOR signaling pathway, a major negative regulator of autophagy [27]. MTOR exists in two functionally distinct complexes, known as mTORC1 and mTORC2, which share two common subunits: mTOR and mLST8. Specific subunits of mTORC1 interact with mTOR to activate it, subsequently inhibiting autophagy [28]. In addition, post‐translational modification of certain subunits has been reported to influence mTOR activity. For instance, acetyl‐CoA derived from peroxisomal β‐oxidation inhibited autophagy by enhancing the acetylation of Raptor (a mTORC1 subunit), leading to the activation of mTOR [29]. AMPK phosphorylated its downstream targets to induce binding to 14‐3‐3 protein, thereby inhibiting mTOR [30]. In the present study, we found that p‐P70 S6K level increased in the hippocampus of P301S mice, indicating mTOR activation. Conversely, Epi treatment resulted in decreased p‐P70 S6K levels, suggesting that autophagy was inhibited in P301S mice but activated by Epi via downregulation of the mTOR signaling pathway.

Based on the data from tau protein degradation tests and mRNA levels, we concluded that the decrease in tau levels induced by Epi was attributed to enhanced tau protein clearance. Tau accumulation primarily occurs due to the impairment of tau degradation via the ubiquitin‐proteasome system and/or the autophagy‐lysosome pathway in tauopathies [31]. When we combined the treatment with proteasome or autophagy inhibitors, we found that CQ or Baf A1 but not MG132 reversed the reduction in tau levels induced by Epi. This finding confirmed that Epi treatment lowered tau levels through the autophagic pathway. To further validate this conclusion, we assessed autophagic markers. Epi treatment resulted in an increased level of LC‐3II and a decreased level of P62, along with a significant increase in LC3‐positive puncta. In addition, using a double‐tagged LC3 approach, we observed a marked increase in autophagy flux. Collectively, these data suggested that Epi treatment promoted the clearance of accumulated tau via the autophagy pathway in P301S transgenic mice.

Autophagy involves induction, autophagosome formation, and lysosome fusion for degradation. Genes linked to neurodegenerative diseases, like tauopathies, can disrupt autophagy at various stages [32]. Phosphatidylinositol‐binding clathrin assembly protein (PICALM) loss inhibits autophagosome formation and maturation in AD [33], while an ALS‐linked mutation in TANK‐binding kinase 1 hinders autophagosome maturation [34]. We also found that human tau accumulation disrupts autophagosome‐lysosome fusion by impairing IST1‐regulated ESCRT‐III complex formation [34]. Compared to the WT control, increased levels of P62 and tau aggregates suggested that autophagic impairments existed in the brains of P301S mice. However, there was no significant change in the level of LC3‐II, nor was there a notable increase in LC3‐positive puncta. The level of LC3‐II protein is regulated by both synthesis and degradation. When we used Baf A1 to inhibit the fusion of autophagosomes and lysosomes—thus inhibiting LC3‐II degradation—LC3‐II significantly increased with Baf A1 treatment. Notably, the increase in LC3‐II in the DMSO versus Baf A1 group of HEK293/Vec was greater than that in the DMSO versus Baf A1 group of HEK293/P301S, suggesting that P301S tau accumulation induced defects in autophagosome formation. Interestingly, rTg4510 mice, which carry another important mutated tau variant, P301L associated with FTD, also exhibit defects in autophagosome formation [35]. Using the same approach, we found that Epi activated autophagy by promoting autophagosome formation.

The 70 kDa ribosomal S6 kinase 1 (p70S6K1) is an important signal transduction enzyme that plays a critical role in cell growth, ‌ protein synthesis, ‌ metabolic regulation, and gene expression. P70S6K1 regulates protein synthesis and cell growth through the phosphorylation of ribosomal protein S6, which serves as the main downstream effector molecule of P70S6K1. The phosphorylation state of S6 is directly associated with cell growth and metabolism. Inhibiting the mTOR pathway, which reduces the phosphorylation of p70S6K1, can promote autophagosome formation [36, 37, 38]. In this study, we found that Epi activated autophagy by inhibiting the mTOR pathway, thereby reducing tau pathology. Notably, the knockout of p70S6K1 has been shown to restore mitochondrial function [39], while P301S overexpression induces energy deficiency due to mitochondrial dysfunction [40, 41]. We speculated that Epi may also restore mitochondrial function through the inactivation of p70S6K1, thus enhancing energy supply and improving cell survival. In addition, Epi decreased tau protein levels without affecting tau mRNA levels in vivo and in vitro, suggesting that Epi does not influence tau gene expression.

Raptor is a specific subunit of mTORC1 that interacts with mTOR to activate it, thereby inhibiting autophagy. Notably, our experimental results showed that Epi treatment inhibited mTOR activity and decreased the level of its upstream protein, Raptor. This led us to speculate that Raptor may be the specific target protein of Epi. By decreasing the Raptor level, Epi may suppress the mTOR pathway, thereby promoting autophagosome formation. Further studies are needed to verify this hypothesis.

Rapamycin can bind to the FK506 binding protein 12 (FKBP12), forming a complex that inhibits the activity of mTOR [42]. As an inhibitor of the mTOR pathway, rapamycin is widely used to promote autophagy in cancers and neurodegenerative diseases [43]. Epi targets the same mTOR pathway as rapamycin, but as a natural product with lower toxicity, it may have more favorable application prospects compared to rapamycin.

In human and animal models, Epi has been shown to improve cognitive functions [44], primarily through mechanisms that reduce oxidative stress and upregulate neuroprotective pathways. In the brains of P301S mice, synaptic dysfunction, neuron loss, and microglial activation are significant contributors to the cognitive deficits associated with tauopathies [45]. Our findings indicated that Epi treatment mitigated neuron loss and spine density reduction while reversing the decreased levels of postsynaptic proteins in the hippocampus of P301S mice. In addition, neuroinflammation was ameliorated, as evidenced by a reduction in activated microglia and astrocytes, along with decreased levels of certain inflammatory factors. Collectively, these effects, along with tau clearance, contributed to the cognitive improvements observed in P301S mice following Epi treatment.

In conclusion, our study provides compelling evidence that Epi, can effectively target the mTOR signaling pathway to promote autophagy and reduce tau pathology in P301S mice. Epi's ability to activate autophagy and enhance the clearance of tau aggregates suggests a potential therapeutic strategy for neurodegenerative diseases characterized by tau accumulation. Furthermore, Epi's mitigation of synaptic and neuronal loss, along with its anti‐inflammatory effects, underscores its multifaceted approach to improving cognitive function in P301S mice. These findings position Epi as a promising candidate for further investigation into the treatment of tauopathies and other neurodegenerative diseases, offering a natural and potentially safer alternative to existing therapeutics.

## 4 Experimental Procedures

### 4.1 Compounds, Reagents, Antibodies, and Plasmids

The compounds, reagents, and antibodies used in the present study are listed in Table S1. Plasmid mCherry‐LC3 was kindly provided by Prof. Xiangnan Zhang (Zhejiang University, China). Plasmid GFP‐RFP‐LC3 was kindly provided by Prof. Qing Tian (Huazhong University of Science and Technology, China). pEGFP‐C1‐P301S‐tau (human tau/tau46 gene mutated at the 301 site Proline to Serine), CFP‐C1‐P301S‐tau (human tau/tau46 gene mutated at the 301 site Proline to Serine), pEGFP‐C1, and CFP‐C1 were purchased from OBIO Biotech (Shanghai, China).

### 4.2 Animals and Drug Administration

Mail P301S transgenic mice [B6; C3‐Tg (Prnp‐MAPT/P301S) PS19Vle/J] and their wild‐type littermates were obtained from the Jackson laboratory. All mice were maintained in a specific pathogen‐free environment, with a 12‐h light/dark cycle, controlled temperature, and ad libitum access to food and water. At 7 months of age, both P301S mice and WT littermates were administered Epi at a dosage of 50 mg/kg via gavage [17, 46], 6 days/week for a duration of 2 months. Control mice received an equal volume of normal saline as a vehicle control. Forty‐five days post‐Epi administration, the mice were treated with CQ at a dose of 50 mg/kg body weight or an equivalent volume of vehicle, delivered daily via intraperitoneal injection for 15 days. CQ was administered intraperitoneally following the daily oral gavage of Epi.

### 4.3 Novel Object Recognition (NOR)

The mice were acclimated to the recognition room for 24 h prior to testing. During the training phase, two identical objects were positioned equidistant from the center of a box measuring 50 × 50 × 50 cm. Each mouse was then placed in the center of the box, facing away from the objects, and allowed to explore freely for 10 min. Interactions, defined as nasal or oral contact with an object, were recorded using a video tracking system. Twenty‐four hours later, one of the objects in the box was replaced with a novel object. The mouse was then returned to the box for another 10 min of free exploration. Results were expressed as discrimination index and preferential index. The preferential index percentage is calculated by new object exploration time / (new object exploration time + old object exploration time) × 100%. The discrimination index was calculated by (new object exploration time − old object exploration time) / (new object exploration time + old object exploration time) × 100%.

### 4.4 Morris Water Maze (MWM)

In accordance with previous reports, the Morris water maze test was conducted to assess the spatial memory and learning ability of mice. The maze, measuring 120 cm in diameter and 60 cm in height, was filled with water maintained at 22 ± 1°C and divided into four quadrants. A platform, measuring 10 cm in diameter and 40 cm in height, was submerged 1.5 cm below the water surface in one of the quadrants. The mice were trained individually to locate the platform over 5 consecutive days, with four trials conducted each day at 30‐min intervals between 1:00 and 6:00 p.m. During the training test, each mouse had a maximum of 60 s to find the platform and was required to remain on it for an additional 30 s. If a mouse failed to find the platform within 60 s, the time was recorded as 60 s, after which the mouse was guided onto the platform and allowed to stay there for 30 s. Twenty‐four hours after the training test, a probe trial was conducted. In this trial, the platform was removed, and the mice were individually placed into the first quadrant opposite to the original platform location. The trajectories of the animals were recorded using Chengdu Taimeng Software Co. Ltd. (China), and relevant parameters were analyzed, including escape latency, the duration spent in the target quadrant, the number of times the animal crossed the location of the previous platform, and swimming speed.

### 4.5 Fear Conditioning Test

The experimental box, measuring 20 cm (length) × 20 cm (width) × 40 cm (height), was outfitted with a ceiling‐mounted video tracking camera, a light, a speaker, a soundproof door, and an electric shock floor. On the first day, the acquisition trial commenced with a 3‐min acclimatization period, each accompanied by a 2‐s, 0.9 mA foot shock, with a 1‐min interval between shocks. To eliminate any residual odors, the box was cleaned with 75% alcohol. The following day, the test was conducted in the same box, using the same tone but without any shocks. Freezing behavior was defined as a complete absence of movement, except for that required for breathing, and was recorded automatically by a video tracking system (Chengdu Taimeng Software Co. Ltd., China).

### 4.6 Cell Culture and Transfection

Human embryonic kidney 293 (HEK293) cells were cultured in DMEM supplemented with 10% fetal bovine serum (FBS). For transfection experiments, HEK293 cells were plated onto 12‐well plates, and plasmids were transfected using the NEOFECT DNA transfection reagent according to the manufacturer's instructions. All cultures were maintained in a humidified incubator at 37°C in an atmosphere containing 5% CO_2_.

### 4.7 Western Blotting

The protein concentration was determined using a BCA kit (Thermo Fisher, NJ, USA). After boiling with loading buffer, 10 or 20 µg of protein extract was separated by 10% or 12% SDS‐PAGE and subsequently transferred to nitrocellulose membranes (Amersham Biosciences, Germany). The membranes were blocked with 5% non‐fat milk for 1 h and then incubated with primary antibodies overnight at 4°C. Following this, the membranes were incubated with IRDyeTM (800CW)‐conjugated anti‐mouse (800 M) or anti‐rabbit IgG (800R) for 1 h at room temperature. Immunoreactive bands were visualized and analyzed using the Odyssey Infrared Imaging System (Licor Biosciences, Lincoln, NE, USA).

### 4.8 Immunohistochemistry and Immunofluorescence

The brains were fixed overnight at 4°C in 4% paraformaldehyde (PFA) for no longer than 24 h. Then the tissue samples were dehydrated using a gradient of ethanol, followed by soaking in xylene and embedding in wax blocks. Subsequently, the samples were cut into 5 µm thick sections using a microtome (Leica RM 2245). Prior to staining, the sections underwent deparaffinization, hydration, and antigen retrieval. Initially, the sections were treated with 3% hydrogen peroxide for 10 to 30 min at room temperature to eliminate endogenous peroxidase activity. After washing with a 0.1% Triton X‐100 PBS solution, the sections were incubated with a 5% BSA solution for 40 min at room temperature. Next, the sections were incubated with primary antibodies overnight at 4°C. Following this, the sections were washed with PBS solution. For immunochemistry, the sections were incubated with secondary and tertiary antibodies at room temperature for 1 h each. Immunoreactivity was developed and visualized using diaminobenzidine. For immunofluorescence, the sections were incubated with a fluorescent secondary antibody (Alexa Fluor 488) for 1 h, sealed with an anti‐fluorescence quencher, and imaged by confocal microscopy (Leica, Wetzlar, Germany).

### 4.9 Imaging and Quantification of LC3 Puncta

The GFP‐RFP‐LC3 and CFP‐C1‐P301S tau plasmids, along with their control vectors, were transfected into HEK293 cells. Fluorescence images were captured using confocal microscopy (Carl Zeiss LSM780), with at least 30 cells imaged in each group. The images were analyzed using Zen software to quantify the number of yellow (GFP^+^RFP^+^ dots, autophagosomes) and red‐only vesicles (GFP^−^RFP^+^ dots, autolysosomes).

### 4.10 Mass Spectrometry Analysis

In brief, hippocampal tissues were lysed to extract proteins, which were then digested with trypsin at 37°C for 14 h. Following digestion, the peptides were desalted and labeled using Tandem Mass Tags (TMTs), then dried and re‐dissolved in 0.1% formic acid (FA) (100 µL) for peptide fractionation. A total of 45 fractions were collected via high‐performance liquid chromatography (HPLC), dried, and re‐dissolved in 0.1% FA (20 µL) for liquid chromatography (LC)‐mass spectrometry (MS)/MS analysis. The acquired data were searched against the UniProt‐Mus musculus database using Proteome Discoverer 2.1 software. Differential expression (DE) of proteins in two groups was set to a threshold of *p* < 0.05. The *p* values for comparisons between groups were calculated using Perseus software (MaxQaunt, Max Planck Institute of Biochemistry, Planegg, Germany). Biological process analysis of DE proteins was conducted using the website of Database for Annotation, Visualization, and Integrated Discovery (DAVID), while pathway analysis was performed using Clue GO software. Visualization of the results was achieved using GraphPad Prism 8.0 and Cytoscape software (3.8.1).

### 4.11 Statistical Analysis

All statistical analyses and result visualizations were performed using GraphPad Prism 8. Data were presented as mean ± SEM. Comparisons between groups were assessed using unpaired Student's *t*‐test, one‐way ANOVA, or two‐way repeated measures ANOVA, followed by Tukey's post hoc test. A *p* value of less than 0.05 was considered statistically significant.

More detailed information about materials and methods is provided in Supporting Information.

## Author Contributions

G.P.L., G.L., X.F.S., and R.M. designed the experiments. Y.Q.W., T.L., and X.J.J. conducted the animal and cell experiments. Y.Q.W., T.L., X.J.J., and J.M.L. performed the Western blotting. T.L., R.M., and Q. L. carried out immunohistochemistry (IHC) and immunofluorescence (IF). X.F.S. and Z.H.Z. conducted the transmission electron microscopy experiments. C.Y.C. and J.M.L. performed Mass spectrometry analysis. Data analysis was carried out by C.Y.C., J.H.Z., and X.F.Y. Y.Q.W., G.L., and G.P.L. wrote the manuscript. All authors have read and approved the final manuscript.

## Ethics Statement

All animal experiments have been reviewed and approved by Ethics Committee of Tongji Medical College, Huazhong University of Science and Technology, with approval number 2021–3192.

## Conflicts of Interest

The authors declare no conflicts of interest.

## Supporting information



Supporting Information

## Data Availability

All mass spectrometry proteomics data have been deposited to the ProteomeXchange Consortium (http://proteomecentral.proteomexchange.org) via the iProX partner repository with the dataset identifier PXD029068.
