# Supplementary material for: (−)‐Epicatechin Rescues Memory Deficits by Activation of Autophagy in a Mouse Model of Tauopathies
Source: MedComm (2020). 2025 Mar 24;6(4):e70144. doi: 10.1002/mco2.70144 (PMC11933444; doi:10.1002/mco2.70144)
Supplement: Supplementary file 1 — Supporting Information [file MCO2-6-e70144-s001.docx]

Supplementary material

(−)-Epicatechin rescues memory deficits by activation of autophagy in a mouse model of tauopathies

**Running title:** (-)-Epicatechin ameliorated tauopathy by autophagy

Yanqing Wu^1,2 #^, Ting Li^3,4 #^, Xingjun Jiang^5 #^, Jianmin Ling^6,7^, **Zaihua Zhao^8^**, Jiahui Zhu^1^, Chongyang Chen^9^, Qian Liu^3^, Xifei Yang^10^, Xuefeng Shen^8*^, Rong Ma^11*^, Gang Li^1*^ and Gongping Liu^3,10,12*^

^1^ Department of Neurology, Union Hospital, Tongji Medical College, Huazhong University of Science and Technology, Wuhan, China

^2^ Health Management Center, Renmin Hospital of Wuhan University, Wuhan, China

^3^ Department of Pathophysiology, School of Basic Medicine, Key Laboratory of Ministry of Education of China and Hubei Province for Neurological Disorders, Tongji Medical College, Huazhong University of Science and Technology, Wuhan, China

^4^ Department of Pathology, Renmin Hospital of Wuhan University, Wuhan, China

^5^ Department of Neurology, The First Affiliated Hospital of Zhengzhou University, Zhengzhou, China

^6^ Department of Emergency Medicine, Tongji Hospital, Tongji Medical College, Huazhong University of Science and Technology, Wuhan, China

^7^ Department of Critical Care Medicine, Tongji Hospital, Tongji Medical College, Huazhong University of Science and Technology, Wuhan, China

^8^ Department of Occupational and Environmental Health and the Ministry of Education Key Lab of Hazard Assessment and Control in Special Operational Environment, School of Public Health, Air Force Medical University, Xi’an, China

^9^ Key Laboratory of Nuclear Medicine, Ministry of Health, Jiangsu Key Laboratory of Molecular Nuclear Medicine, Jiangsu Institute of Nuclear Medicine, Wuxi, China

^10^ Key Laboratory of Modern Toxicology of Shenzhen, Shenzhen Center for Disease Control and Prevention, Shenzhen, China

^11^ Department of Pharmacology, School of Basic Medicine, Tongji Medical College, Huazhong University of Science and Technology, Wuhan, China

^12^ Co-innovation Center of Neuroregeneration, Nantong University, Nantong, China

**Supplementary Figures**


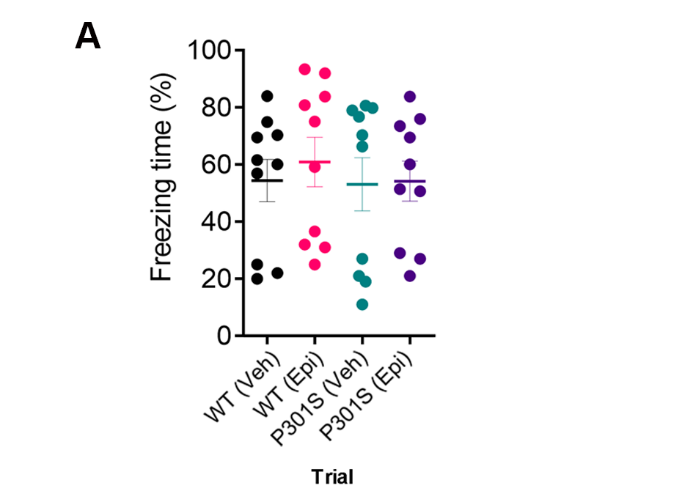


**Figure S1. There was no difference in the freezing time during training by fear condition test.**

The freezing time of the mice during training was detected by fear condition test. (n=10 mice for each group). All data are shown as mean ± SEM, one-way ANOVA test followed by Tukey’s post hoc test.

WT (Veh): wild type mice treated with vehicle; WT (Epi): wild type mice treated with (−)-Epicatechin; P301S (Veh): P301S mice treated with vehicle; P301S (Epi): P301S mice treated with (−)-Epicatechin.


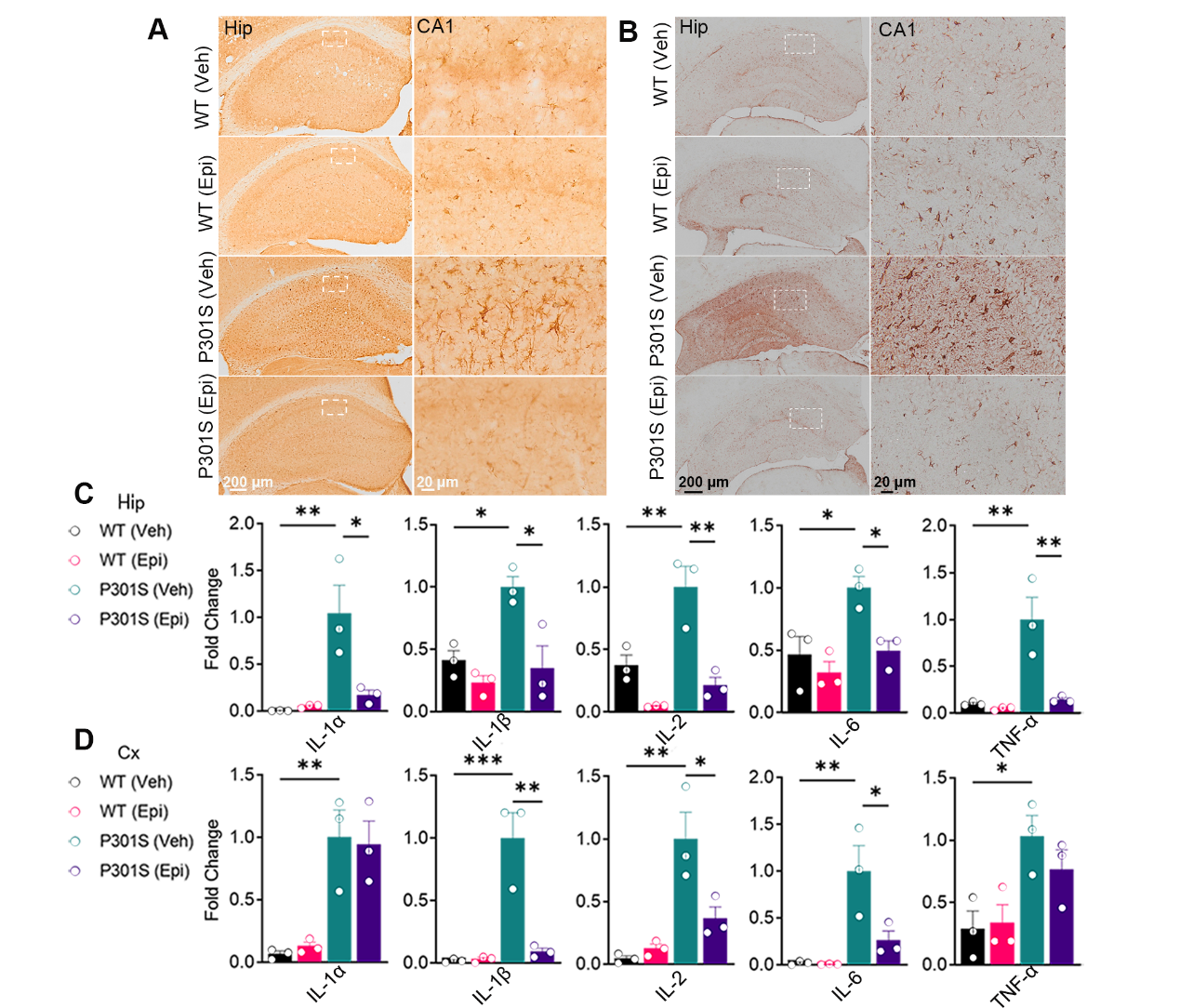


**Figure S2. Epi treatment attenuated neuroinflammation**

**(A)** Representative images of IBA1 staining in the hippocampus (Hip). **(B)** Representative images of GFAP staining in the hippocampus. **(C, D)** The mRNA levels of interferon (IL-1α, IL-1β, IL-2, IL-6) and TNF-α of the hippocampus (Hip) **(C)** or cortex (Cx) **(D)** were detected by qPCR (n = 3 for each group, One-way ANOVA test followed by Tukey’s post hoc test. ***, *p <* 0.05, ****, *p <* 0.01, ***, *p <* 0.001). All data are shown as mean ± SEM.


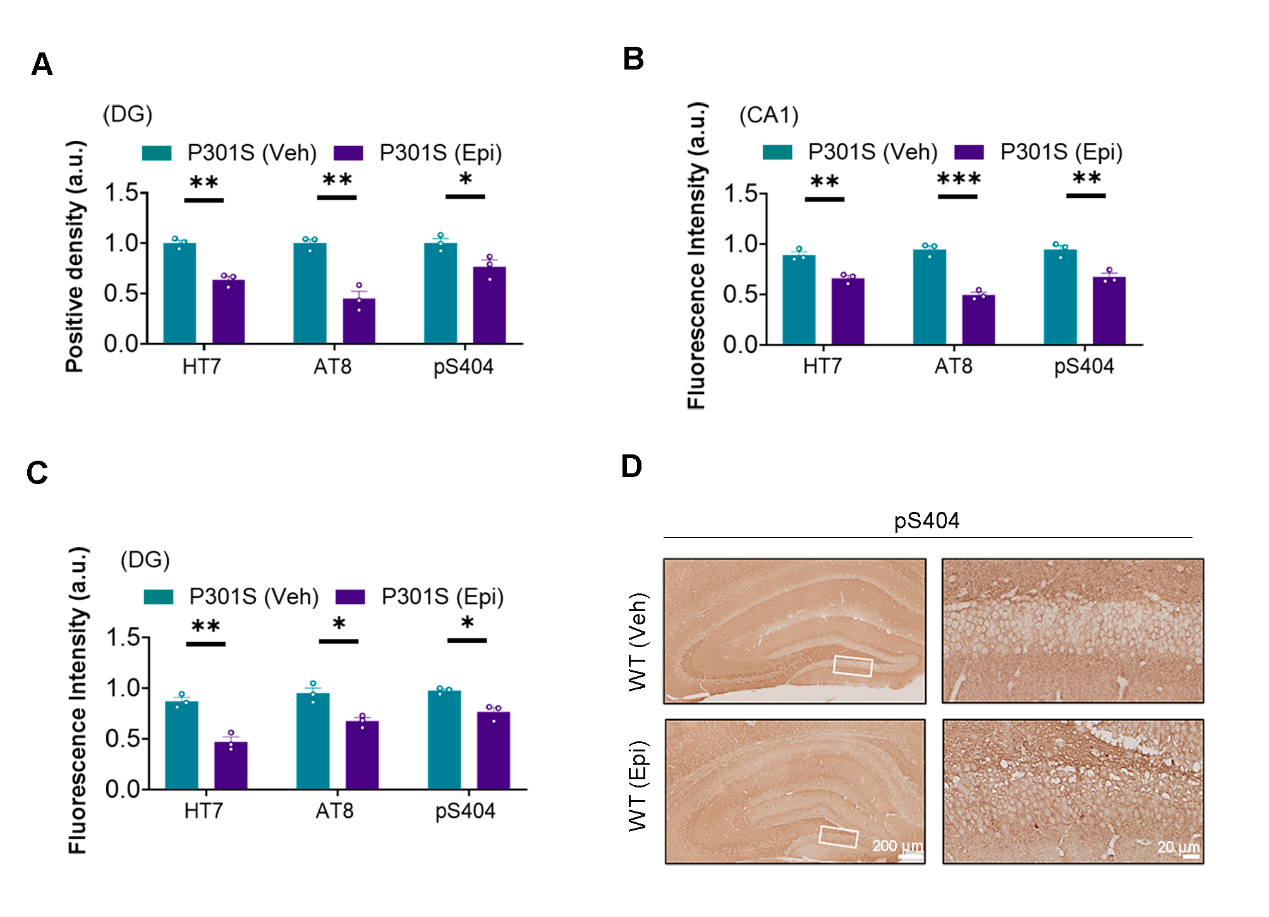


**Figure S3. Epi treatment reduced tau pathology in the hippocampus of P301S mice.**

**(A)** Quantitative analysis of immunohistochemistry for HT7, AT8, and pS404 in the DG region, the representative images shown in Figure 3I. **(B, C)** Quantitative analysis of immunofluorescence for HT7, AT8, and pS404 in the CA1 **(B)** and DG **(C)** region, the representative images shown in Figure 3J. **(D)** Representative immunohistochemical images of pS404 in the hippocampus of WT (Veh) and WT (Epi) mice. All data are shown as mean ± SEM, n = 3 for each group. Unpaired t-test for all. *, p < 0.05, **, p < 0.01, ***, p < 0.001, vs. P301S (Veh).

WT (Veh): wild type treated with vehicle, WT (Epi): wild type treated with (−)-Epicatechin. P301S (Veh): P301S mice treated with vehicle; P301S (Epi): P301S mice treated with (−)-Epicatechin.


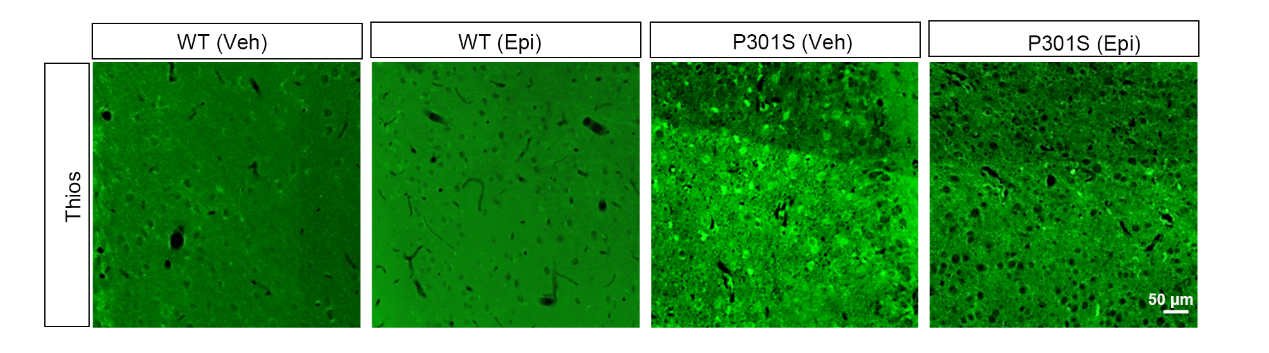


**Figure S4. Epi treatment reversed tau aggregation in the piriform cortex of P301S mice**

Representative images of Thioflavin S (Thios) staining in the piriform cortex of the mice.


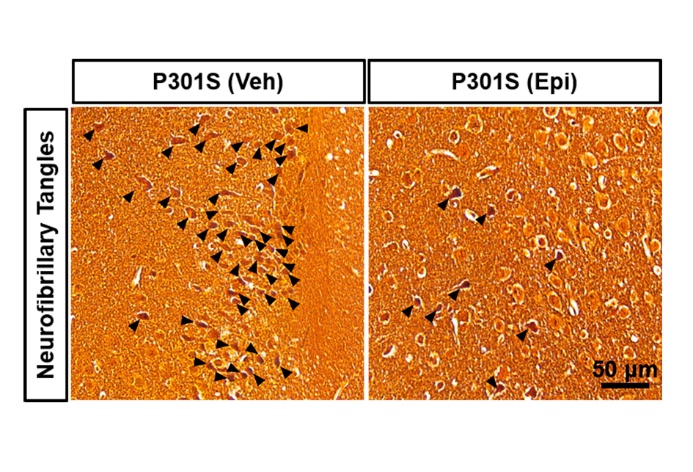


**Figure S5. Epi treatment attenuated neurofibrillary tangles in the piriform cortex of P301S mice**

Representative images of Silver staining in the piriform cortex of the mice. The black triangle represents the entanglement of neurofibrillary tangles.

P301S (Veh): P301S mice treated with vehicle; P301S (Epi): P301S mice treated with (−)-Epicatechin.


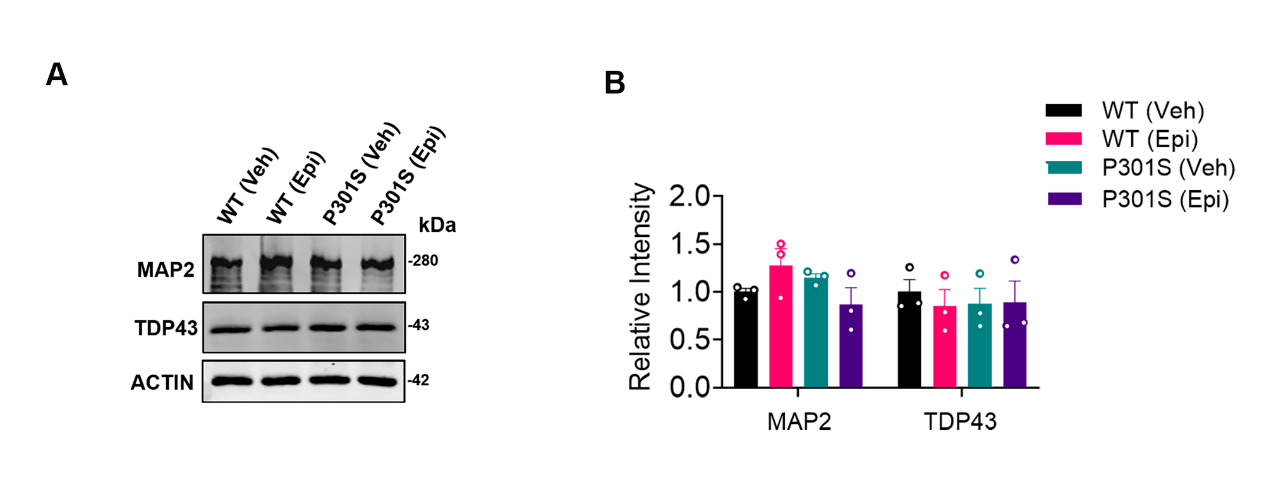


**Figure S6. Epi treatment did not alter MAP2 and TDP43 protein levels.**

**(A, B)** WT and P301S mice were treated with Epi for 2 months, hippocampi (Hip) were lysed and MAP2 and TDP43 were detected by western blotting.

All data are shown as mean ± SEM, n = 3 for each group. One-way ANOVA test followed by Tukey’s post hoc test.


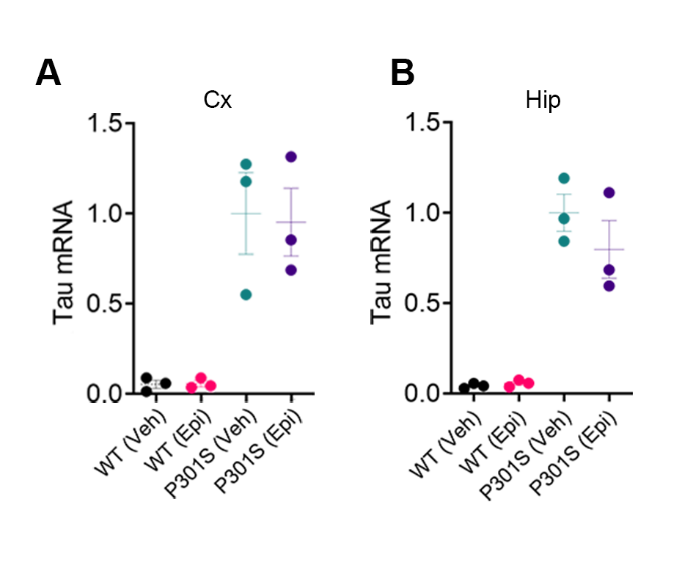


**Figure S7.** **The mRNA level of tau** **did not change during Epi treatment.**

**(A, B)** Administration with Epi had no effects on the mRNA level of tau in the cortex **(**Cx, **A)** or hippocampus **(**hip, **B)** of P301S mice. n = 3 for each group, one-way ANOVA test followed by Tukey’s post hoc test. All data are shown as mean ± SEM.


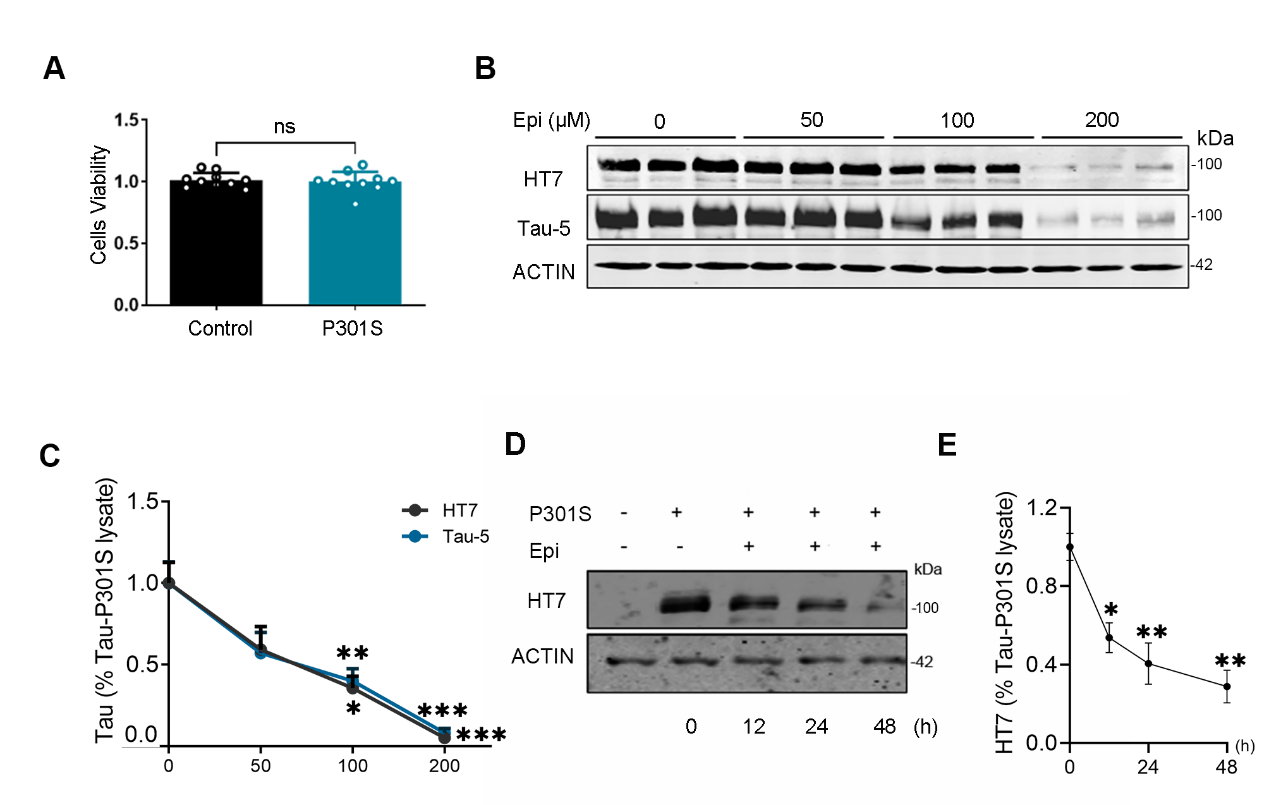


**Figure S8.** **Epi reduced total tau protein in a dose dependent manner.**

**(A)** P301S-htau transfection had no significant effect in cell viability. n = 10. **(B, C)** HEK293/P301S (HEK293 cells transiently transfected with P301S-tau) were treated with Epi (0, 50, 100, 200 μM) for 24 h, and total tau (recognized by HT7 and Tau-5) level was detected by Western blotting (***, *p <* 0.05, ****, *p <* 0.01, ***, *p <* 0.001 *vs* 0 μM Epi). **(C, D)** HEK293/P301S cells were treated with Epi (100 μM) for 12, 24 or 48 h, and total tau (recognized by HT7) level was detected by western blotting (***, *p <* 0.05, ****, *p <* 0.01 *vs* 0 h). All data are shown as mean ± SEM, n = 3 for each group. One-way ANOVA test followed by Tukey’s post hoc test.


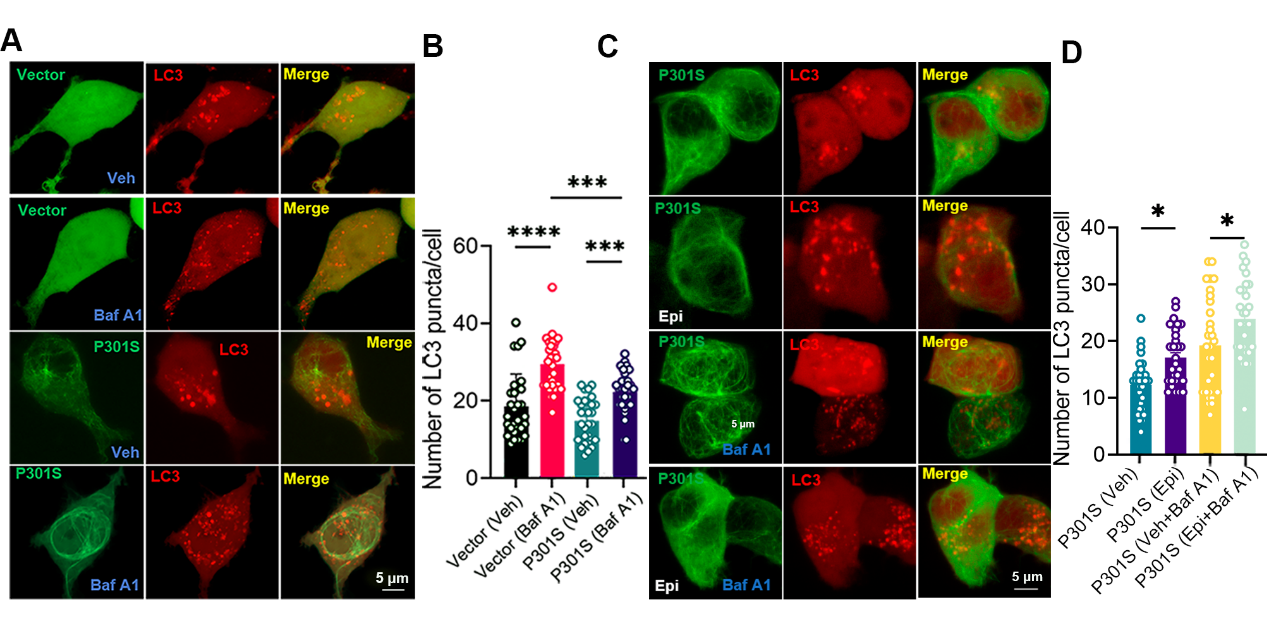


**Figure S9.** **Administration with Epi increased LC3-II level and LC3-positive puncta**

**(A, B)** HEK293 cells co-transfected with mCherry-LC3 and P301S tau (EGFP-C1-P301S) or the vector (EGFP-C1) were treated with Baf A1 (100 μM) or vehicle for 24 h, LC3-positive puncta was measured by direct fluorescence (at least 30 cells were analyzed for each group). **(C, D)** HEK293/P301S cells transfected with mCherry-LC3 were treated with Epi (100 μM), Baf A1 (100 μM) or Epi and Baf A1 for 24 h, LC3-positive puncta was measured by direct fluorescence (at least 30 cells were analyzed for each group). All data are shown as mean ± SEM. One-way ANOVA test followed by Tukey’s post hoc test. *, *p* < 0.05, ***, *p* < 0.001, ****, *p* < 0.0001.


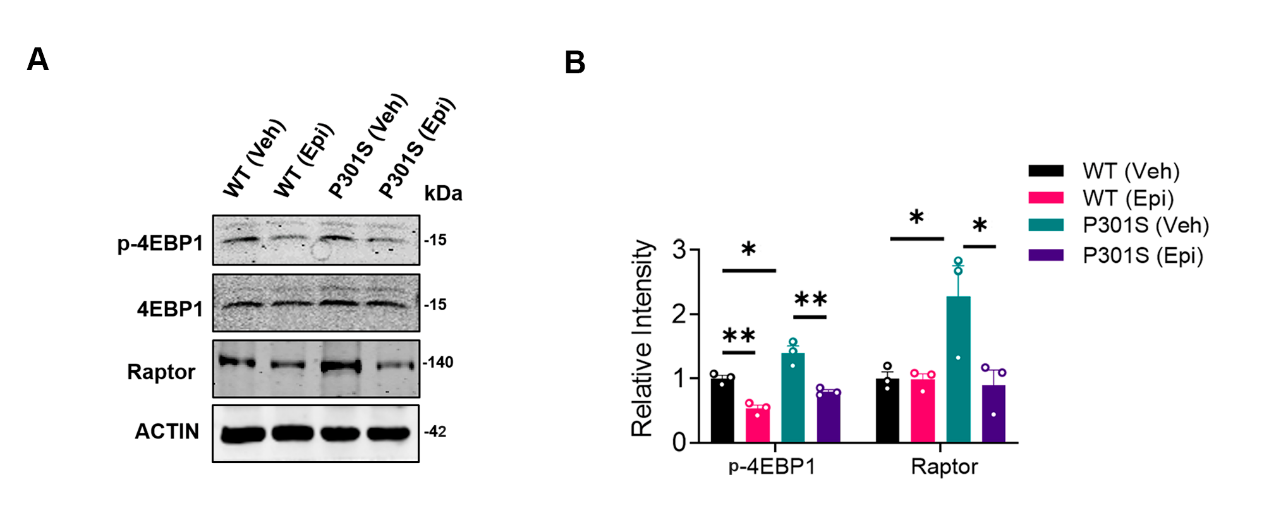


**Figure S10.** **Epi treatment inactivated mTOR**

**(A, B)** WT and P301S mice were treated with Epi for 2 months. The hippocampus (Hip) was lysed, and phosphorylated 4EBP1 (p-4EBP1) and Raptor were detected by western blotting. All data are shown as mean ± SEM, n = 3 for each group. One-way ANOVA test followed by Tukey’s post hoc test. *, p < 0.05, **, p < 0.01.

**Supplementary Materials and Methods**

**Extraction of soluble and insoluble tau**

Hippocampal or frontotemporal cortex tissues were homogenized in RIPA buffer (Beyotime, P0013B) containing a 1×Protease Inhibitor Cocktail on ice. The homogenates were centrifuged at 13,000 × g for 20 min at 4°C, and the supernatants were collected as the RIPA fraction, designated as the soluble tau fraction. The pellet was then homogenized twice, followed by centrifugation at 13,000 × g for 20 min at 4°C prior to resuspension. It was then incubated with agitation in a 1:1 (w:v) solution of 70% formic acid at 4°C overnight. The formic acid suspension was centrifuged at 18,000 × g for 20 min at 4°C, and the supernatant was collected. After evaporating the formic acid, the precipitates were dissolved in 2 × SDS-PAGE sample buffer, representing the insoluble tau fraction.

**Thioflavin S, Nissl and Golgi staining**

Five-micrometer brain sections were washed 3 times in 1×TBS, with each wash lasting 5 min. The sections were then incubated in fresh/filtered 0.3% Thioflavin S (dissolved in 50% ethanol) at room temperature for 10 min in the dark. After incubation, the sections were decolorized in 50% ethanol three times for 5 minutes each and subsequently washed in 1× TBS for 5 minutes in the dark. Finally, the sections were dried in the shade and sealed with Glycerol PBS Mounting Tablet. The slides with 10 μm sections were stained with Nissl staining solution (Servicebio, GP1043) for 2-5 min and then washed by running water. The slides were dried and coverslipped with **Permount TM Mounting Medium.**

Dendritic spines of neurons in the hippocampus of animals were studied using the FD Rapid GolgiStain™ kit (FD NeuroTechnologies, USA). The brain tissues were immersed in equal volumes of Solutions A and B and stored at room temperature in the dark for 4 weeks. They were then transferred to Solution C and kept in the dark for an additional week. The brain tissues were sliced into 100 µm thick sections, which were rinsed twice with Milli-Q water for 4 min each and incubated in a mixture of Solution D and Solution E for 10 min. After dehydration with ethanol and clearing in xylene, the sections were coverslipped with Permount.

**Silver Staining**

Neurofibrillary tangles were visualized using the Glycine Silver Staining Kit (G1052-500T, servicebio). The experiment was conducted strictly according to the provided instructions. Paraffin brain sections were first immersed sequentially in Xylene I and II for 20 min. They were then subjected to a gradient of alcohol (100%, 100%, 90%, 75%), with each step lasting 5 min. Following this, the sections were washed several times with tap water and distilled water. Next, the sections were incubated in Glycine silver staining solution C for 5 min, followed by three washes with distilled water. They were then transferred to Glycine silver staining solution B for 5 min. Afterward, the brain sections were removed and any residual Glycine silver staining solution B was quickly shaken off the tissue. The sections were then transferred to Glycine silver staining solution A I (preheated to 45 °C) for a few seconds, and subsequently placed into Glycine silver staining solution A II (also preheated to 45 °C) for a few seconds. Finally, the sections were washed with distilled water, dehydrated, cleared with absolute ethanol and xylene, and then mounted with neutral gum.

**RNA isolation and Quantitative real-time PCR**

Total RNA was extracted from brain tissue using Trizol (TaKaRa). The RNA was then reverse transcribed and amplified with the the PrimeScript^TM^RT reagent kit with gDNA Eraser (Takara) and One-Step SYBR PrimeScript PLUS RT-PCR Kit (TaKaRa). Quantitative RT-PCR (qRT-PCR) was performed and analyzed using the ABI Step one plus Real-Time PCR System (Applied Biosystems). The mRNA levels of the target genes were normalized to β-actin mRNA expression levels and calculated using the 2^-ΔΔCt^ method for relative expression analysis. Primer sequences are listed in Table S2.

**Transmission electron microscopy**

The number of autophagosomes was quantitatively analyzed using electron microscopy. Briefly, mice were perfused with PBS (pH 7.4), followed by a fixation solution containing 2.5% glutaraldehyde and 4% paraformaldehyde in phosphate buffer (PB, pH 7.4). The brains were then removed and post-fixed for 2 h in the same fixative at 4 ℃. After fixation, the brains were sectioned into 50 μm thick slices. The entire hippocampus was selected and further processed by post-fixation in 1% osmium tetroxide (dissolved in 0.1 M PB) for 25 min. The samples were then stained with 1% uranyl acetate, dehydrated in graded ethanol, and embedded in epoxy resin. Ultrathin sections (60 nm) were prepared and then stained with lead citrate before imaging with a JEM-1400 electron microscope (JEOL LTD, Tokyo, Japan).

**Table S1.** **The compounds, regents and antibodies used in the study**

| Category | Source | Cat Number |
| --- | --- | --- |
| Mouse monoclonal anti-Tau (HT7) antibody | Thermo Fisher, USA | Cat#MN1000 |
| Mouse monoclonal anti-Tau-5 antibody | Abcam, USA | Cat#ab80579 |
| Rabbit polyclonal anti-Tau (Phospho-Ser396) antibody | Signalway Antibody, USA | Cat#11102 |
| Rabbit polyclonal anti-Tau (Phospho-Ser404) antibody | Signalway Antibody, USA | Cat#11112 |
| Rabbit polyclonal anti-Tau (Phospho-Ser262) antibody | Signalway Antibody, USA | Cat#11111 |
| Mouse monoclonal anti-Human PHF-tau (AT8) antibody | Thermo Fisher, USA | Cat#MN1020 |
| Rabbit monoclonal anti-LC3B antibody | Abcam, USA | Cat#ab51520 |
| Mouse monoclonal anti-P62 antibody | Abcam, USA | Cat#**ab56416** |
| Rabbit polyclonal anti-mTOR antibody | Cell Signaling Technology, USA | Cat#2972 |
| Rabbit polyclonal anti-P70 S6K antibody | Proteintech, China | Cat#14485-1-AP |
| Rabbit polyclonal anti-p-P70 S6K antibody | Cell Signaling Technology, USA | Cat#9204 |
| Rabbit monoclonal anti-Beclin1 antibody | Abcam, USA | Cat#ab210498 |
| Rabbit monoclonal anti-Atg5 antibody | Abcam, USA | Cat#ab108327 |
| Rabbit monoclonal anti-Atg12 antibody | Abcam, USA | Cat#ab52472 |
| Rabbit polyclonal anti- PSD95 antibody | Abcam, USA | Cat#ab18258 |
| Rabbit monoclonal anti-synaptophysin antibody | Abcam, USA | Cat#ab32127 |
| Rabbit polyclonal anti-SYN1 antibody | Proteintech, China | Cat#20258-1-AP |
| Rabbit monoclonal anti-GluN1 antibody | Abcam, USA | Cat# ab109182 |
| Rabbit monoclonal anti-GluN2A antibody | Abcam, USA | Cat# ab124913 |
| Rabbit monoclonal anti-GluN2B antibody | Abcam, USA | Cat# ab254356 |
| Rabbit polyclonal anti-Iba1 antibody | Wako, Japan | Cat#019-19741 |
| Mouse monoclonal anti-GFAP antibody | Cell Signaling Technology, USA | Cat#3670 |
| Mouse monoclonal anti-Actin antibody | Abcam, USA | Cat# ab8226 |
| Mouse monoclonal anti-DM1A antibody | Abcam, USA | Cat# ab7291 |
| Mouse monoclonal anti-GAPDH antibody | Proteintech, China | Cat#60004-1-AP |
| Rabbit polyclonal anti-TDP43 antibody | Proteintech, China | Cat#10782-2-AP |
| Rabbit monoclonal anti-Raptor antibody | Cell Signaling Technology, USA | Cat#2280T |
| Rabbit monoclonal anti-P-4EBP1 antibody | Cell Signaling Technology, USA | Cat#2855S |
| Mouse monoclonal anti-4EBP1 antibody | Proteintech, China | Cat#60246-1-Ig |
| Rabbit polyclonal anti-MAP2 antibody | Sigma-Aldrich, USA | Cat#AB5622 |
| (-)-Epicatechin (Epi) | Sigma-Aldrich, USA | Cat#E1753 |
| Chloroquine (CQ) | Sigma-Aldrich, USA | Cat#C6628 |
| Cycloheximide (CHX) | Med Chem Express, USA | Cat#HY-12320 |
| Mg132 | Med Chem Express, USA | Cat#HY-13259 |
| Bafilomycin A1 (Baf A1) | Abcam, USA | Cat# ab120497 |
| Thioflavin S | Sigma-Aldrich, USA | Cat# T1892 |
| Nissl dye solution | Wuhan Servicebio, China | Cat# G1036 |
| NEOFECTTM DNA transfection reagent | Beijing NEOFECT, China | Cat#TF20121201 |

**Table S2. Oligonucleotides used in the study**

| qPCR | Primer sequences (5'to3') |
| --- | --- |
| Human 4R tau Forward | GAAGCTGGATCTTAGCAACG |
| Human 4R tau Reverse | GACGTGTTTGATATTATCCT |
| Mouse IL-1α Forward | CGAAGACTACAGTTCTGCCATT |
| Mouse IL-1α Reverse | GACGTTTCAGAGGTTCTCAGAG |
| Mouse IL-1β Forward | GCACTACAGGCTCCGAGATGAA |
| Mouse IL-1βReverse | GTCGTTGCTTGGTTCTCCTTGT |
| Mouse IL-2 Forward | GTGCTCCTTGTCAACAGCG |
| Mouse IL-2 Reverse | GGGGAGTTTCAGGTTCCTGTA |
| Mouse IL-6 Forward | AGTGGCTAAGGACCAAGAC |
| Mouse IL-6 Reverse | ATAACGCACTAGGTTTGCCGA |
| Mouse TNFα Forward | CACGCTCTTCTGTCTACTGAACTTC |
| Mouse TNFα Reverse | ATGATCTGAGTGTGAGGGTCTGG |
| Mouse Actin Forward | GGCTGTATTCCCCTCCATCG |
| Mouse Actin Reverse | CCAGTTGGTAACAATGCCATGT |
